# In Situ Electrochemical Activation of Pseudo‐Layered NbS_3_ via Interlayer Expansion and Dual Redox for High Mg‐Ion Storage

**DOI:** 10.1002/advs.74690

**Published:** 2026-03-07

**Authors:** Pengcheng Jing, Atsushi Inoishi, Chengcheng Zhao, Eiichi Kobayashi, Peng Ren, Isaac Abrahams, Duncan H. Gregory

**Affiliations:** ^1^ WestCHEM School of Chemistry Joseph Black Building University of Glasgow Glasgow UK; ^2^ Tianmushan Laboratory Hangzhou China; ^3^ Institute for Materials Chemistry and Engineering Kyushu University Kasuga Fukuoka Japan; ^4^ Kyushu Synchrotron Light Research Center Tosu Saga Japan; ^5^ Department of Chemistry Queen Mary University of London London UK

**Keywords:** magnesium ion batteries, niobium trisulfide, interlayer expansion, dual redox mechanism, 1‐butyl‐1‐methylpyrrolidinium cations

## Abstract

Magnesium‐ion batteries (MIBs) are a promising alternative to lithium‐ion technologies due to their inherent safety and potential for sustainable, large‐scale energy storage, yet their development remains hindered by the scarcity of high‐capacity cathode materials. In this study, we  reveal a significant leap in performance in a structurally unique quasi‐1D pseudo‐layered NbS_3_ cathode for MIBs, achieved through electrochemical interlayer engineering. *In operando* and ex situ PXRD, SEM‐EDS, and XPS confirm the intercalation of 1‐butyl‐1‐methylpyrrolidinium (BMPyrr^+^) cations, which results in substantial expansion of the interlayer spacing. This expansion not only enhances magnesium ion diffusion but also activates the dual Nb^4+^/Nb^3+^ and S_2_^2−^/S^2−^ redox processes for access to abundant ion storage sites, as elucidated by ex situ XAS analysis. In addition, multiple coupled factors, including BMPyrr^+^‐enabled channel opening and electrochemically induced morphological reconfiguration (i.e., nanosizing/fragmentation) further promote pseudo capacitive behavior. Consequently, the expanded NbS_3_ electrode delivers a high reversible capacity of up to 200 mA h g^−1^ at 50 mA g^−1^, plus excellent cycling stability, significantly outperforming its unmodified counterpart. This work highlights NbS_3_ as a novel dual‐redox trichalcogenide cathode for MIBs and demonstrates the power of interlayer expansion in unlocking inherent redox reactions for improving performance in multivalent‐based energy storage systems.

## Graphical abstract

A quasi‐1D pseudo‐layered NbS_3_ cathode for magnesium‐ion batteries can be created through electrochemically induced interlayer expansion. The widened ion‐transport channels enable fast magnesium ion diffusion and unlock coupled cationic‐anionic redox chemistry. Together with in situ nanosizing, this strategy delivers markedly enhanced capacity and cycling stability, highlighting NbS_3_ as a promising multivalent‐storage cathode.

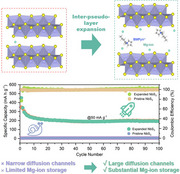

## 1 Introduction

Magnesium ion batteries (MIBs) have emerged as one of the most promising post‐lithium energy storage technologies due to their inherent safety, natural abundance, and high volumetric capacity of magnesium metal [1, 2, 3, 4]. Unlike lithium and sodium metal anodes, magnesium metal does not form dendrites in many electrolyte systems under typical cycling conditions [3, 4]. Nevertheless, the advancement of MIBs remains constrained by several challenges, chief among them being the scarcity of high‐performance, reversible cathode materials. This limitation is largely attributed to the strong electrostatic interactions between divalent Mg^2+^ ions and the lattice host, which severely impede ion diffusion and limit the number of electrochemically active sites [3, 5].

A major breakthrough was achieved in 2000 when Aurbach and co‐workers demonstrated reversible magnesium storage in Chevrel phase Mo_6_S_8_ [6]. Since then, extensive efforts have been devoted to exploring cathode materials that enable effective magnesium ion insertion, encompassing oxides (e.g., V_5_O_12_·6H_2_O and MoO_3_), chalcogenides (e.g., MoS_2_, CoNi_2_S_4_, and CuS), and polyanions (e.g., VOPO_4_) [7, 8, 9, 10, 11, 12]. Transition metal chalcogenides (TMCs) with (pseudo‐)layered or chain‐like architectures, such as MoS_2_, VS_2,_ and VS_4_ [9, 13, 14], have attracted significant attention due to their tunable interlayer or interchain spacing and relatively weak electrostatic interactions with magnesium ions. In particular, the van der Waals (vdW) gaps in such structures provide accessible pathways for magnesium ion transport, especially when combined with interlayer engineering strategies.

Inspired by their success in lithium‐ion systems, interlayer expansion has been adopted in MIBs to overcome kinetic limitations. Theoretical and experimental studies have confirmed that increasing interlayer spacing can reduce magnesium ion intercalation barriers and diffusion energies, while also increasing the number of accessible redox sites. For instance, density functional theory (DFT) simulations showed that interlayer‐widened MoS_2_ exhibits significantly lower energy barriers to both magnesium ion intercalation and migration [15]. Experimentally, interlayer‐expanded MoS_2_ achieved reversible capacities of 80–100 mA h g^−1^, twice to four times higher than the 20–50 mA h g^−1^ typical of its unmodified counterparts [16, 17]. A range of interlayer expansion approaches has been demonstrated, including chemical pre‐intercalation and electrolyte‐derived guest insertion [13, 18, 19]. For example, interlayer‐expanded CuS has been achieved via intercalation of the bulky organic cation cetyltrimethylammonium (CTA^+^) during solvothermal synthesis, delivering improved magnesium storage [20]. By contrast, under electrochemical conditions, in situ intercalation of bulky organic cations, such as 1‐butyl‐1‐methylpyrrolidinium (BMPyrr^+^), has been shown to enlarge interlayer spacings in layered TMCs during initial discharge, thereby enhancing magnesium diffusion and storage. For example, BMPyrr^+^‐induced expansion increased the interlayer distance of TiS_2_ from ∼0.57 to 1.86 nm, enabling appreciable reversible capacity [21]. A similar effect was observed in our recent work on vanadium‐substituted MoS_2_, where BMPyrr^+^ intercalation unlocked latent magnesium storage functionality [22]. However, most of these dichalcogenide‐based systems rely solely on transition metal redox, limiting their ability to accommodate multi‐electron transfer and thereby lowering magnesium storage capacities.

To address this, increasing attention has been paid to cathode materials capable of exploiting both cationic and anionic redox processes [23, 24]. For instance, BMPyrr^+^‐expanded VS_4_ electrodes activated both V and S redox couples [25], delivering a high initial discharge capacity of *∼*665 mA h g^−1^ at a current density of 50 mA g^−1^, far exceeding that of pristine VS_4_ (150–251 mA h g^−1^) [26, 27]. Quasi‐1D transition metal trisulfides, such as NbS_3_, represent a structurally unique and underexplored class of redox‐active chalcogenides. These materials consist of 1D [MS_6_]*_n_* chains (M = transition metal) arranged into pseudo‐layers separated by weak van der Waals interactions. The presence of disulfide (S_2_^2−^) moieties within the chains provides a platform for engaging anionic redox, potentially enabling dual cationic/anionic redox chemistry. Moreover, the anisotropic structure facilitates rapid electron transport along the chain axis while permitting 2D magnesium ion diffusion between pseudo‐layers [28, 29]. Despite their structural advantages and proven utility in lithium‐ion batteries [30, 31], transition metal trisulfides remain largely underexplored in MIBs.

In this work, we report a significant magnesium ion storage enhancement in triclinic quasi‐1D pseudo‐layered NbS_3_ by employing an interlayer engineering strategy. By introducing BMPyrr^+^ from the electrolyte as a pillaring agent, interlayer expansion is achieved in situ, substantially enhancing magnesium ion diffusion kinetics and unlocking reversible redox activity. The expanded NbS_3_ cathode delivers stable reversible capacities up to 200 mA h g^−1^ with excellent cyclability at a current density of 50 mA g^−1^, whereas the unmodified electrode exhibits negligible capacity. *In operando* powder X‐ray diffraction (PXRD), ex situ SEM‐EDS, and X‐ray photoelectron spectroscopy (XPS) confirm the structural expansion induced by BMPyrr^+^ intercalation during initial discharge. Moreover, ex situ XPS and X‐ray absorption spectroscopy (XAS) verify the activation of Nb^4+^/Nb^3+^ and S_2_^2−^/S^2−^ redox couples following structural expansion. These findings underscore the dual benefits of interlayer engineering and redox activation in achieving high‐performance MIB cathodes and highlight the untapped potential of transition metal trisulfides in multivalent energy storage systems.

## 2 Results and Discussion

**SCHEME 1:**
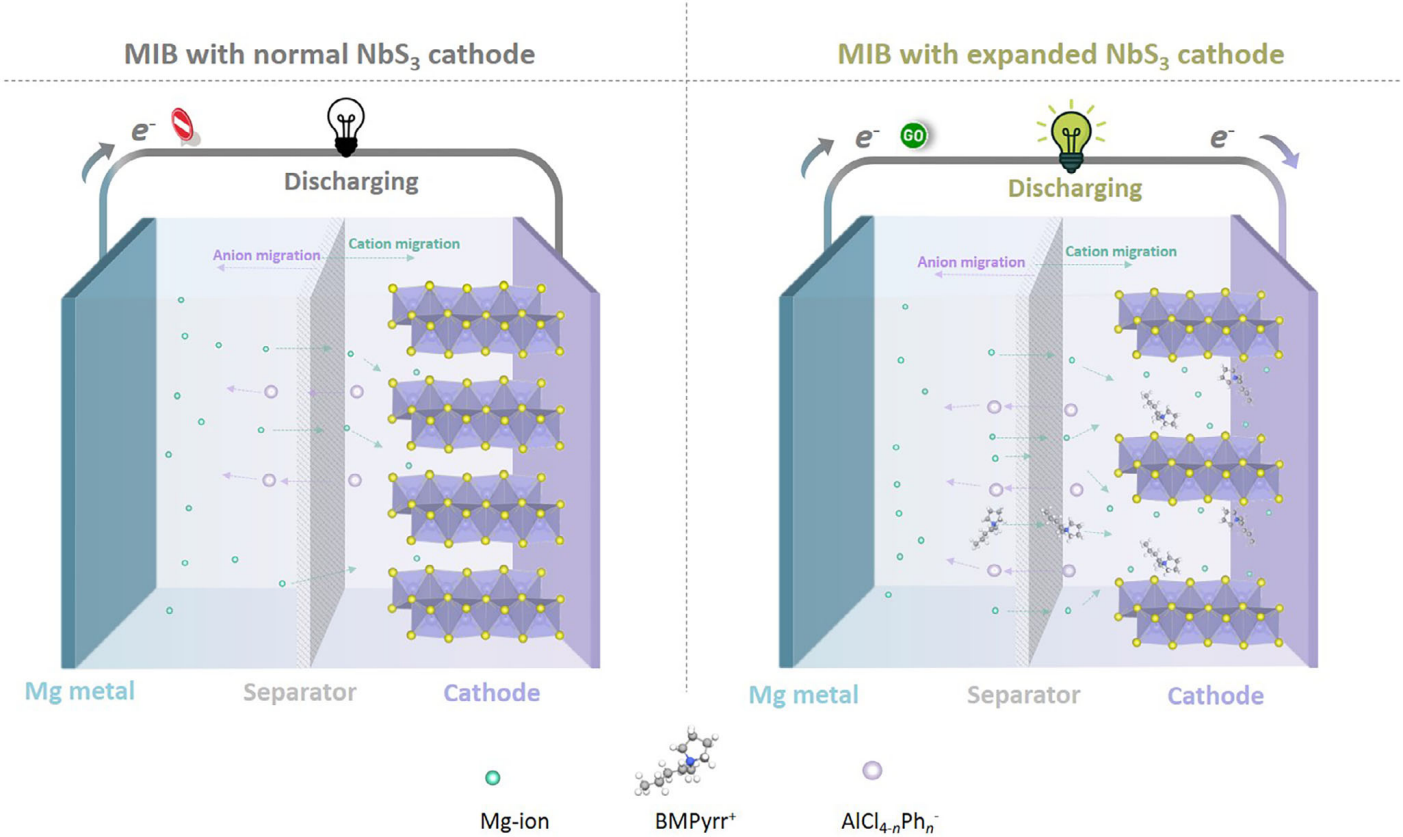
Illustration of MIB batteries employing two NbS_3_ cathode configurations: (a) pristine NbS_3_ featuring narrow transport galleries that restrict Mg^2+^ diffusion and limit the utilization of ion‐storage sites, and (b) expanded NbS_3_ with enlarged vdW channels that facilitate faster Mg^2+^ migration and activate previously inaccessible redox‐active sites. Both schematics represent the cells under discharge conditions.

As illustrated in Scheme 1, a typical Mg‐NbS_3_ cell consists of a Mg metal anode, an NbS_3_ cathode, and a glassfiber separator infused with a liquid magnesium electrolyte. Pristine NbS_3_ features a quasi‐1D pseudo‐layered framework, where the tightly packed chains give rise to narrow vdW channels and thus unfavorable electrostatic interactions with Mg^2+^. These strong host‐ion interactions significantly restrict ion transport, leading to sluggish diffusion kinetics and limited accessible storage sites under native structural constraints. Strategically expanding the interlayer spacing can effectively mitigate these kinetic bottlenecks. By widening the transport galleries, the intercalant‐lattice interactions are weakened, and the energy barrier for Mg^2+^ insertion and migration is lowered, enabling full activation of latent redox‐active sites that remain inaccessible in the pristine phase. Meanwhile, the intrinsic S_2_^2−^/S^2−^ redox centers within NbS_3_ provide an additional contribution pathway, facilitating enhanced charge transfer and improved capacity. In this study, we introduce bulky BMPyrr^+^ cations to induce structural expansion, following the proven applicability of this additive in promoting interlayer widening across (pseudo‐)layered sulfides and oxides as well as low‐dimensional chain‐type sulfides.

**FIGURE 1:**
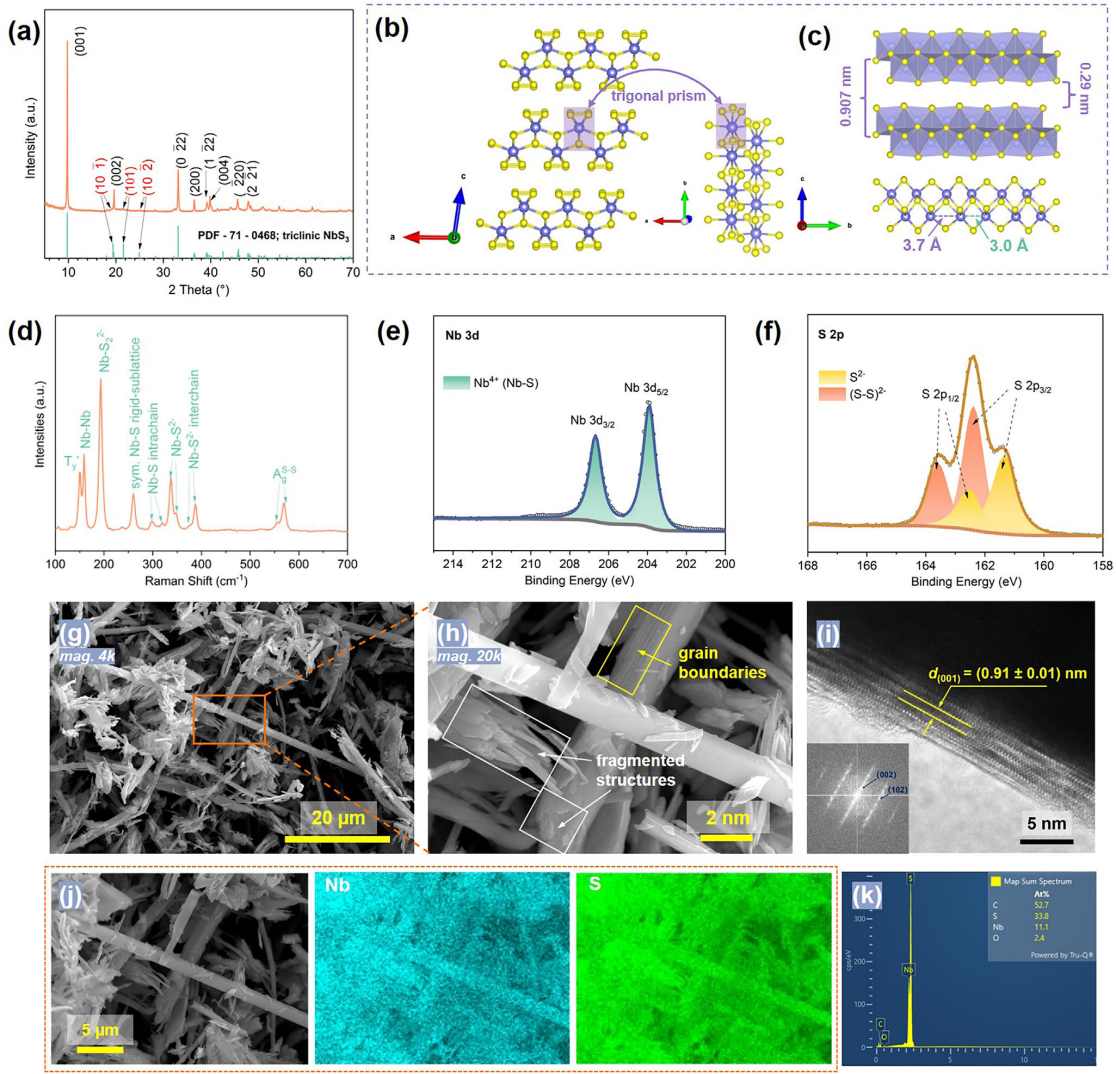
Material characterisation of the as‐synthesised NbS_3_. (a) PXRD pattern compared with the standard PDF card for the triclinic phase (PDF ‐ 71 ‐ 0468). Crystal structure of triclinic NbS_3_ projected onto (b) the *ac* and *ab* planes, and (c) *bc* plane, highlighting [NbS_6_] trigonal prisms (light violet), interlayer spacing (*∼*0.907 nm), vdW gap (*∼*0.29 nm), and alternating long (3.7 Å) and short (3.0 Å) Nb–Nb distances. (d) Raman spectrum of NbS_3_. High‐resolution XPS results showing the (e) Nb 3d and (f) S 2p regions of the spectrum. SEM images at (g) low and (h) high magnifications. (i) HRTEM image with corresponding fast Fourier transformation (FFT) pattern (inset). (j) SEM‐EDS elemental maps of Nb and S, and (k) the corresponding EDS spectrum.

The NbS_3_ compound was synthesized via a simple physical vapor transport (PVT) reaction between elemental niobium and sulfur. The phase purity of the as‐obtained NbS_3_ powder was initially assessed by powder X‐ray diffraction (PXRD) in Bragg‐Brentano geometry. As shown in Figure 1a, all diffraction peaks match well with the triclinic NbS_3_ phase (PDF ‐ 71 ‐ 0468, space group *P*‐1), confirming phase purity. The pronounced (00*l*) reflections suggest a strong preferred orientation along the *c*‐axis. Notably, reflections from (*h*0*l*) planes are largely absent, which is likely due to stacking disorder along the *c*‐axis induced by lateral displacement of pseudo‐layers along the *a*‐axis, a phenomenon also reported previously for NbS_3_, MoS_2_, and WS_2_ [32, 33, 34]. The crystal structure of triclinic pseudo‐layered NbS_3_ is illustrated in Figure 1b,c. It consists of [NbS_6_] trigonal prisms (light violet) connected via polar covalent S–Nb–S bridges along the *b*‐axis to form infinite 1D chains. These [NbS_6_]*_n_* chains align along the *a*‐axis and assemble into pseudo‐layers held together by weaker interchain Nb‐S interactions. The pseudo‐layers are further stacked along the *c*‐axis via vdW forces (Figure 1c), resulting in the formation of bulk crystal. The interlayer spacing derived from the (001) reflection is *∼*0.907 nm, which corresponds to a vdW gap of *∼*0.29 nm. Similar to monoclinic VS_4_ [25, 35], NbS_3_ exhibits alternating long (3.7 Å) and short (3.0 Å) Nb–Nb distances along the chain direction (*b*‐axis), which can be attributed to Peierls distortion arising from a half‐filled conduction band [32, 36].

Raman spectroscopy further confirms the structural features of NbS_3_ (Figure 1d). Ten prominent vibrational modes are observed at ∼151, 159, 193, 260, 298, 319, 337, 348, 387, and 569 cm^−1^, consistent with previously reported values [37, 38, 39]. Specifically, the low‐frequency peak at 151 cm^−1^ corresponds to a rigid‐chain T_y_^′^ mode resulting from lattice contraction along the chain direction [39, 40]. The adjacent peak at 159 cm^−1^ is assigned to in‐plane Nb–Nb stretching. The most intense peak at 193 cm^−1^ is attributed to Nb‐S_2_^2−^ stretching, while the band at 260 cm^−1^ corresponds to a rigid symmetrical valence Nb–S vibration within the sublattice. The two peaks at 298 and 319 cm^−1^ are ascribed to Nb–S intrachain modes. The overlapping peaks at 337 and 348 cm^−1^ are associated with Nb‐S^2−^ vibrations, although the exact interchain/intrachain origin remains unclear. The adjacent doublet at 377 and 387 cm^−1^ likely represents interchain Nb–S^2−^ vibrations. Additionally, the high‐frequency peak at 569 cm^−1^ originates from S–S oscillation within disulfide moieties (S_2_^2−^).

The chemical composition and oxidation states of Nb and S were further analyzed via XPS. As shown in Figure 1e, the Nb 3d high‐resolution spectrum presents a pair of peaks at *∼*203.9 eV (Nb 3d_5/2_) and 206.7 eV (Nb 3d_3/2_), characteristic of Nb^4+^ in NbS_3_ [41, 42]. In the S 2p region (Figure 1f), two doublets are identified. The first, at 162.4 eV (2p_3/2_) and 163.6 eV (2p_1/2_), is assigned to disulfide (S_2_^2−^) species, while the second, at 161.3 and 162.5 eV, corresponds to S^2−^ [38, 43, 44, 45]. These results confirm the coexistence of high‐valence Nb^4+^ cations and S_2_^2−^ anions, supporting the potential for dual anionic and cationic redox processes in NbS_3_.

Morphological characterization was carried out using SEM‐EDS and TEM. The low‐magnification SEM image (Figure 1g) reveals a mixture of thick fibre‐like particles (20–100 µm long, 1–3 µm wide) and shorter rods or ribbon‐like crystallites (2–20 µm long, 0.1–1 µm wide). Closer inspection of the cross region of the fibers and rods/ribbons (Figure 1h) highlights the grain boundaries (highlighted in yellow) and fragmented pieces (white box), likely resulting from mechanical cleavage during sample handling. The HRTEM image, together with the corresponding indexed fast Fourier transform (FFT) pattern (Figure 1i), confirms an interlayer spacing of 0.91 ± 0.01 nm, consistent with the PXRD‐derived spacing of 0.907 nm. Elemental mapping by SEM‐EDS (Figure 1j) shows homogeneous distributions of Nb and S throughout the particles. The atomic ratio of S to Nb is 3.1, as determined from the EDS spectrum (Figure 1k), in good agreement with the expected NbS_3_ stoichiometry.

**FIGURE 2:**
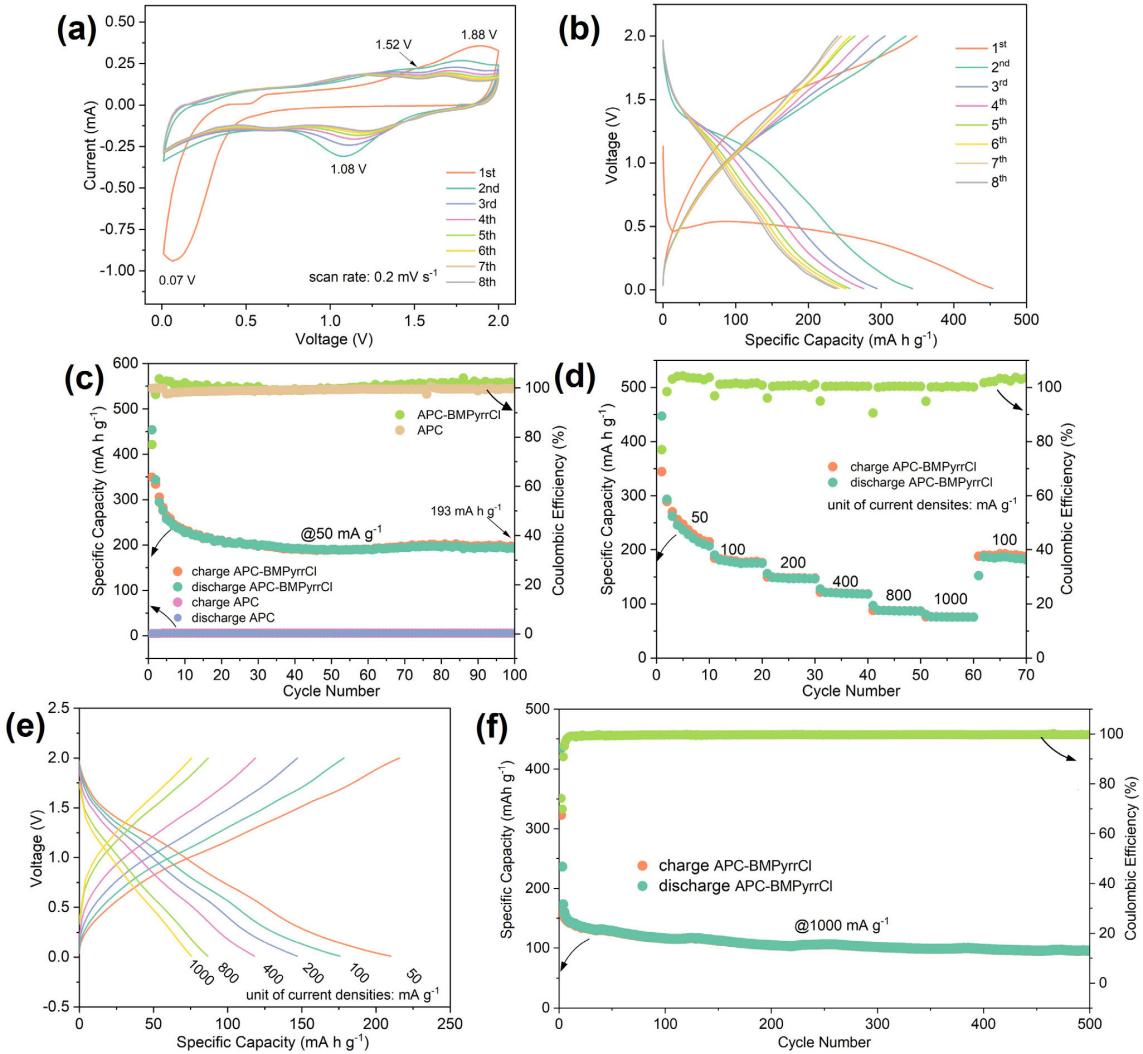
Electrochemical behavior and performance of NbS_3_ electrodes in BMPyrrCl‐containing APC electrolyte: (a) CV curves over the initial 8 cycles at a scan rate of 0.2 mV s^−1^. (b) Galvanostatic (dis)charge profiles at a current density of 50 mA g^−1^. (c) Low‐current‐density (50 mA g^−1^) cycling performance. (d) Rate capability at various current densities (values indicated in the plot), and (e) corresponding selected (dis)charge curves at different current densities. (f) Long‐term cycling performance at a high current density of 1000 mA h g^−1^.

The electrochemical behavior of the NbS_3_ electrode was primarily evaluated through cyclic voltammetry (CV) and galvanostatic (dis)charge tests, with particular attention to the effect of BMPyrrCl additive in the electrolyte. In the absence of BMPyrrCl, the CV curves of the NbS_3_ electrode (Figure S1a) reveal only two broad and weak cathodic features at ∼0.52 V and 0.24 V during the initial scan, while no discernible redox features are observed in subsequent scans, indicative of poor reversibility and limited magnesium storage. In stark contrast, the addition of BMPyrrCl significantly enhances electrochemical activity. As shown in Figure 2a and Figure S1b, the first cathodic scan exhibits a weak peak centered at 0.57 V and a broad intense peak at 0.07 V (spanning 0.5–0.01 V), corresponding to early BMPyrr^+^‐associated channel opening and subsequent substantial magnesium ion intercalation (with possible co‐insertion of BMPyrr^+^), respectively (See Figure 4 for mechanistic support). This activation is likely facilitated by BMPyrr^+^‐induced interlayer expansion, similar to BMPyrr^+^‐expanded VS_4_ [26]. The corresponding anodic scan displays two broad overlapping peaks at ∼1.52 V and 1.88 V, albeit with lower current. These results suggest that part of the intercalated species, including BMPyrr^+^ and magnesium ions, remain trapped within the lattice, rendering the first cycle partially irreversible. From the second cycle onward, new and more defined redox couples emerge at ∼1.08/1.78 V and ∼0.63/1.38 V, respectively, gradually converging over the first several cycles. This trend reflects a decrease in polarization and a transition into a more stable reaction regime. The declining peak currents over the initial six cycles indicate capacity fading, likely due to the structural evolution or partial degradation of the active material, which is further explored below. The galvanostatic (dis)charge profiles obtained at a current density of 50 mA g^−1^ (Figure 2b) further confirm the activation behavior observed in the CV analysis. The first discharge curve features a quasi‐plateau at around 0.5 V followed by a sloping region, delivering a high initial capacity of 454 mA h g^−1^, slightly higher than the theoretical value (425 mA h g^−1^) for three electron transfer. This phenomenon has also been observed in previous reports [46], and may be attributed to additional surface charge storage induced by in situ nanosizing, partial decomposition of the active materials, and resulting side reactions with the electrolyte, which will be analyzed furtherbelow. The corresponding charge process yields ∼349 mA h g^−1^, corresponding to an initial Coulombic efficiency of 77%. The subsequent (dis)charge curves exhibit sloping characteristics and gradually stabilize, consistent with an electrochemically accessible and reconfigured NbS_3_ host framework.

The cycling performance of the NbS_3_ electrode in BMPyrrCl‐containing APC is presented in Figure 2c. After the initial activation and a modest capacity decline over the first 10 cycles, the electrode retains a reversible capacity of ∼193 mA h g^−1^ after 100 cycles. In contrast, the same electrode tested in pure APC electrolyte shows negligible capacity (∼5 mA h g^−1^), highlighting the critical role of BMPyrr^+^ in activating substantial magnesium diffusion and storage likely by a means of interlayer expansion. The NbS_3_ electrode exhibits good rate capability (Figure 2d), delivering ∼210, 175, 147, 118, 87, and 75 mA h g^−1^ at escalating current densities of 50, 100, 200, 400, 800, and 1000 mA g^−1^, respectively. These values are competitive with those reported for structurally related 1D metal sulfide hosts such as VS_4_ in MIBs [26, 47, 48, 49, 50]. The corresponding representative (dis)charge profiles at various current densities are presented in Figure 2e. In addition, the NbS_3_ electrode was subjected to prolonged high‐rate cycling at 1000 mA g^−1^. As illustrated in Figure 2f, the electrode maintains a reasonable capacity of 96 mA h g^−1^ after 500 cycles with relatively stable operation. Future improvements in rate capability may be attained by improving the electrical conductivity of the electrode through in situ compositing conductive materials such as graphene or carbon nanotubes, as demonstrated in related chalcogenide systems including VS_4_‐carbon nanotubes, CuS‐graphene, and TiS_2_‐MXene composites in MIBs [50, 51, 52].

**FIGURE 3:**
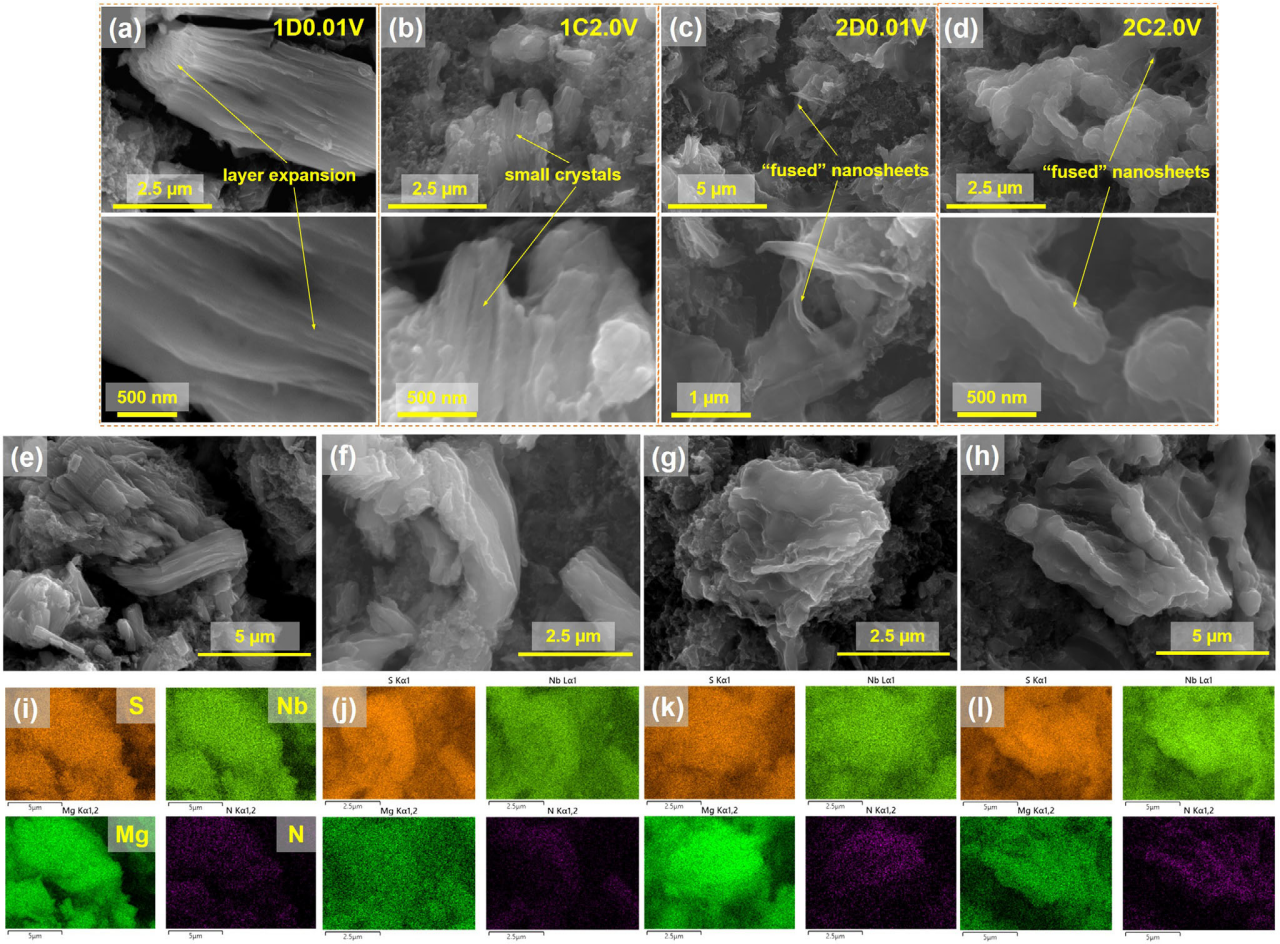
(a—d) SEM morphological features and (e)—(l) SEM‐EDS elemental maps of S, Nb, Mg, and N at various states of charge: (e, i) 1D0.01 V; (f, j) 1C2.0 V; (g, k) 2D0.01 V; and (h, l) 2C2.0 V.

To elucidate the microstructural and compositional evolution of NbS_3_ electrodes upon cycling, ex situ SEM and EDS mapping were conducted at various charge/discharge states. As shown in Figure 3a, after the first discharge, the pristine rod‐like morphology of NbS_3_ transforms dramatically. The longitudinal surfaces exhibit pronounced wrinkling, while the cross‐sections become notably expanded and loosened. These morphological changes suggest that the intercalation of bulky BMPyrr^+^ cations induces interlayer expansion, leading to partial exfoliation or swelling perpendicular to the pseudo‐layer stacking direction. Upon the first charge (Figure 3b), the structure evolves into thin, wrinkled sheets, presumably due to strain fluctuation resulted from magnesium ion de‐intercalation. After the second (dis)charge cycle (Figure 3c,d), the bulk particles are fully converted into loosely stacked, wrinkled nanosheets, with fused or crumpled morphologies resembling thermally treated materials. This nanosizing process is associated with BMPyrr^+^‐enabled channel opening in the initial cycle and may further evolve via mechanical fragmentation during repeated magnesium ion (de)intercalation in subsequent cycles. This dynamic morphology likely contributes to improved ion‐transport kinetics and sustained cyclability, while excessive fragmentation may also partially contribute to early‐cycle capacity decay.

To correlate these microstructural changes with compositional dynamics, SEM‐EDS elemental mapping was performed at four key (dis)charge states (Figure 3e–i). Sulfur, niobium, magnesium, and nitrogen were found to be homogeneously distributed across particle regions. Notably, the Mg signal increases after discharge and decreases upon charge, confirming reversible magnesium ion (de‐)intercalation. In contrast, the nitrogen signal remains relatively stable throughout, implying that BMPyrr^+^ cations, once intercalated, remain largely trapped within the lattice and act as permanent “pillars”. Quantitative EDS analysis (Figures S2 and S3; and Table S1) shows that Mg/Nb and Cl/Nb atomic ratios vary reversibly with cycling, suggesting a mixed Mg^2+^ and Mg*_x_* Cl*_y_* ^+^ intercalants. The largely constant Al/Nb ratio implies that Al and a portion of Cl derive from surface‐adsorbed electrolyte residues [18]. The S/Nb atomic ratio drops from 3 to around 2 after cycling, indicating sulfur loss. This degradation is likely a key contributor to the capacity decay observed during the initial cycles and reflects a broader challenge in MIB chemistry: the susceptibility of transition metal sulfides to degradation in halide‐containing electrolytes. A similar phenomenon has been reported or observed for other metal sulfides, such as VS_4_ and TiSe_2_, in chloride‐rich MIB electrolytes [21, 26, 53, 54]. While corrosive Cl^−^ species may partly account for this instability, the fundamental cause likely lies in the high charge density of Mg^2+^, which imposes greater electrostatic strain on the host lattice than monovalent Li^+^. This may destabilize sulfur bonds and trigger irreversible decomposition pathways. For instance, although a three‐electron redox process involving S_2_^2–^/S^2–^ and Nb^4+^/Nb^3+^ is accessible in both Li and Mg systems, sulfur degradation is only evident in the MIB context. DFT simulations by Arsentev et al., showed that Mg^2+^ intercalation beyond Mg_0.5_TiS_3_ can induce lattice collapse and irreversible structural degradation [55]. Consistently, SEM‐EDS analysis of cycled electrodes reveals sulfur‐rich regions segregated from Nb‐rich domains (Figure S4), suggestive of sulfur‐containing decomposition products (e.g., MgS_x_‐like species). However, direct identification of these products and their transport pathways will rely on future deeper mechanistic understanding of degradation pathways using direct speciation‐level proof such as *in operando* Raman, NMR and/or online electrochemical mass spectrometry (OEMS), which would further enable rational mitigation strategies to improve performance by suppressing structural degradation and decomposition. These may include structural modifications to reinforce the host lattice, hybridization with conductive frameworks to improve interfacial stability, or the use of chloride‐free, Mg‐compatible electrolytes to suppress side reactions. Despite these degradation‐related limitations, the NbS_3_ cathode already delivers competitive achievable gravimetric and volumetric energy densities (ca. 295 W h kg^−1^ and 1221 W h L^−1^, respectively) among representative MIB cathodes (Figure S5 and Table S2). Therefore, mitigating sulfur‐loss pathways provides a clear route toward further improving practical energy output.

**FIGURE 4:**
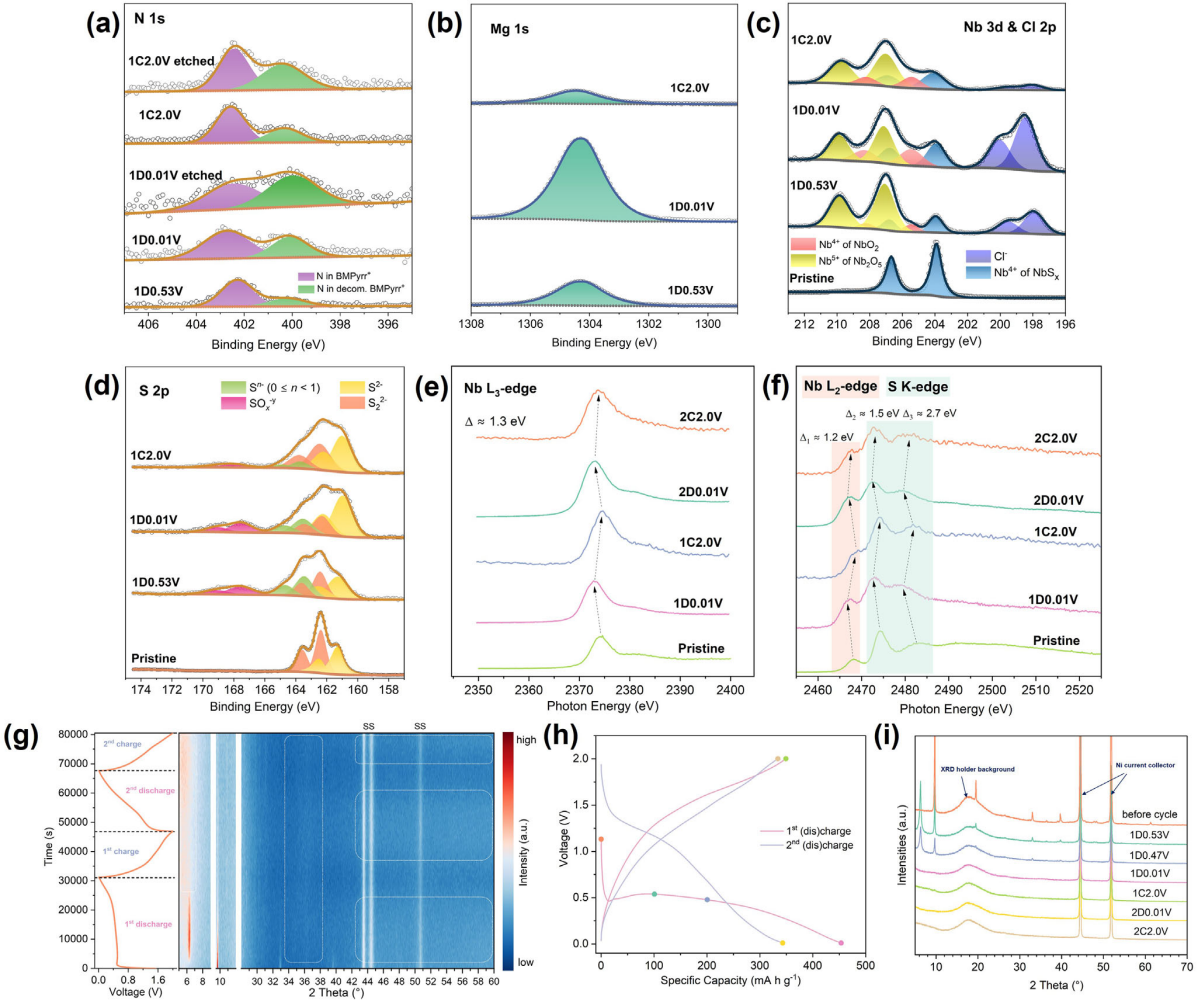
Spectroscopic characterization of the NbS_3_ electrodes at various (dis)charge states: High‐resolution XPS regions of (a) N 1s, (b) Mg 2p, (c) Nb 3d and Cl 2p, and (d) S 2p transitions. XAS spectra at the (e) Nb L_3_‐edge and (f) Nb L_2_‐ and S K‐edges in total fluorescence yielding mode. (g) Initial (dis)charge curves (left side) at 80 mA g^−1^ and corresponding contour plot of *in operando* PXRD patterns (right side). Peaks from the stainless‐steel sample cell are denoted as SS. (h) Galvanostatic (dis)charge curves and corresponding (i) *ex situ* PXRD patterns. Solid circles with various colors in (h) represent the selected (dis)charge states corresponding to the PXRD patterns in (i).

The chemical evolution of the NbS_3_ electrode during cycling, including the presence of N, Mg, Cl, and the oxidation states of Nb and S, was investigated using ex situ XPS. Figure 4a and Figure S6 show that N 1s signals, originating exclusively from BMPyrr^+^, emerge at early discharge (∼0.53 V) and increase slightly toward 0.01 V, while the Mg 1s signal increases markedly at later discharge states, indicating initial BMPyrr^+^ insertion followed by overlapping insertion behavior at deeper discharge states. The N 1s signal persists during charging to 2.0 V, and remains detectable after Ar^+^ etching, supporting that BMPyrr^+^ intercalates within NbS_3_ and acts as a structural pillar during subsequent cycling. Figure 4b,c display the continuous growth of the Mg 1s peak and Cl 2p peaks during discharge, followed by replenishment upon charging. This reversible variation is indicative of the (de‐)intercalation of Mg^2+^/Mg*_x_* Cl*_y_* ^+^ species, consistent with the EDS quantitative results and published MIB research on VS_2_ and VS_4_ [14, 18]. Mg–Cl carrier species are known to exhibit faster transport than bare Mg^2+^ due to reduced electrostatic penalties. However, this kinetic advantage becomes effective only after interlayer channels are sufficiently opened. In our NbS_3_ system, negligible reversible magnesium storage is observed before structural expansion, even though Mg–Cl carriers are already present in the electrolyte, whereas substantial reversible capacity is obtained after BMPyrr^+^‐enabled channel opening. These observations distinguish the roles of BMPyrr^+^ (channel‐opening/pillaring) and Mg‐based chloride carriers (transport species). The above results confirm that BMPyrr^+^ intercalation and interlayer expansion facilitate the intercalation and diffusion kinetics of magnesium‐based intercalants. In the Nb 3d region (Figure 4c), upon discharging to 1D0.01 V, the characteristic doublet peaks of Nb^4+^ in NbS_3_ at ∼203.9/206.8 eV decrease in intensity, while peaks corresponding to Nb^4+^ and Nb^5+^ in Nb oxides appear. This indirectly suggests the reduction of Nb^4+^ to lower Nb^3+^ in NbS_3_, which likely underwent oxidation to NbO_2_ and Nb_2_O_5_ during sample washing and transferring for measurements, a phenomenon commonly observed in air‐sensitive intercalated chalcogenides [56]. The charged electrode encountered a similar surface oxidation issue, which will be clarified in subsequent XAS analysis. In the S 2p spectra, the two peaks for S^2−^ strengthen upon discharge, while the S_2_^2−^ signal weakens, consistent with the reduction of S_2_^2−^ to S^2−^. After charging, the S_2_^2−^ component partially recovers, suggesting a reversible S_2_^2−^/S^2−^ redox process. However, the presence of heavily oxidized sulfur species, including S*^n–^* (0 ≤ *n* < 1) and SO*_x_^y–^* further manifests the surface oxidation of the cycled electrodes.

To further verify the redox mechanisms, total fluorescence yielding XAS measurements were performed to examine the oxidation states of Nb and S at different stages of cycling, providing chemical information at a larger depth (∼100 nm) under airtight conditions. Figure 4e,f shows the Nb L‐edge and S K‐edge spectra of NbS_3_ electrodes at different (dis)charge states. In the pristine NbS_3_ electrode, the Nb L‐edge comprises of L_3_ and L_2_ peaks at ∼2374.3 and 2468.1 eV, corresponding to the dipole‐allowed transitions from Nb 2p_3/2_ and 2p_1/2_ to 4d orbitals, respectively. Meanwhile, the S K‐edge spectrum exhibits a pre‐edge peak at ∼2474.2 eV, corresponding to transitions from 1s to 3p orbitals [57], and a broad peak at ∼2482.7 eV, associated with transitions to higher‐energy hybridised orbitals (S 3p/Nb 5s) [58]. Upon the first discharge, Nb L‐edge peaks shift to lower photon energies of ∼2373.0 and 2466.9 eV, suggesting an increase in electron density around Nb and a reduction in effective nuclear charge, consistent with the reduction of Nb^4+^ to Nb^3+^, as supported by the Faradaic capacity equivalent to three electrons per NbS_3_ and previous literature reports [46]. Similarly, the shift of S K‐edge peaks to lower energies (∼2472.7 and 2480.0 eV) upon discharging confirms the reduction of S_2_^2−^ species to S^2−^, consistent with previous XPS analysis and literature [46, 59, 60]. After the first charge, both Nb L‐edge and S K‐edge peaks almost fully recover. In subsequent cycles, similar shifts in the Nb L‐edge and S K‐edge peaks are observed, confirming the stability and reversibility of the dual cationic (Nb^4+^/Nb^3+^) and anionic (S_2_^2−^/S^2−^) redox reactions during cycling. The continuous evolution of these spectra highlights the dynamic changes in the electronic structure of the NbS_3_ electrode and corroborates the XPS findings, establishing the reversible dual redox mechanism as a key factor in the activated magnesium ion storage performance.

The structural changes of the NbS_3_ electrode during cycling were further investigated by *in operando* and ex situ PXRD analysis. The contour plot of the *in operando* PXRD patterns, together with the corresponding (dis)charge curves, is presented in Figure 4g. Upon the first discharge, a new diffraction peak emerges at a lower 2*θ* value of ∼6.3°, replacing the original NbS_3_ peak at ∼9.7°. According to Bragg's law, the interlayer spacing increases from *ca*. 0.907 to 1.393 nm, corresponding to an increased vdW gap (defined as the distance between the top and bottom sulfur layers of two adjacent NbS_3_ pseudo‐layers) of 0.776 nm, consistent with the estimated length of BMPyrr^+^ (0.7–0.8 nm). This result, together with XPS analysis, supports intercalation and stable pillaring of BMPyrr^+^ in the expanded structure [21, 22, 26, 61, 62]. Such structural expansion facilitates magnesium‐ion intercalation/diffusion and enables dual Nb^4+^/Nb^3+^ and S_2_^2−^/S^2−^ redox for enhanced ion storage. The absence of higher‐angle peaks corresponding to the expanded phase may be attributed to an increased preferred orientation along the *c*‐axis and/or a reduction in crystallinity during the intercalation process. Upon further discharge to the cutoff voltage, the peak associated with the expanded phase vanishes, signaling the onset of amorphization due to continued BMPyrr^+^/magnesium ion co‐intercalation. In subsequent cycles, the PXRD peaks for both the pristine and expanded phases barely recover, with the diffraction pattern showing only broad, weak intensity regions (highlighted by white dotted lines), indicating a transition toward a largely amorphous state. Ex situ PXRD measurements (Bragg‐Brentano geometry, reflection mode) in Figure 4h,i also validate the structural evolution of the NbS_3_ electrode during various discharge and charge states, suggesting expanded poorly crystalized structures (*a‐phase*) over repeated magnesium ion (de‐)intercalation.

**FIGURE 5:**
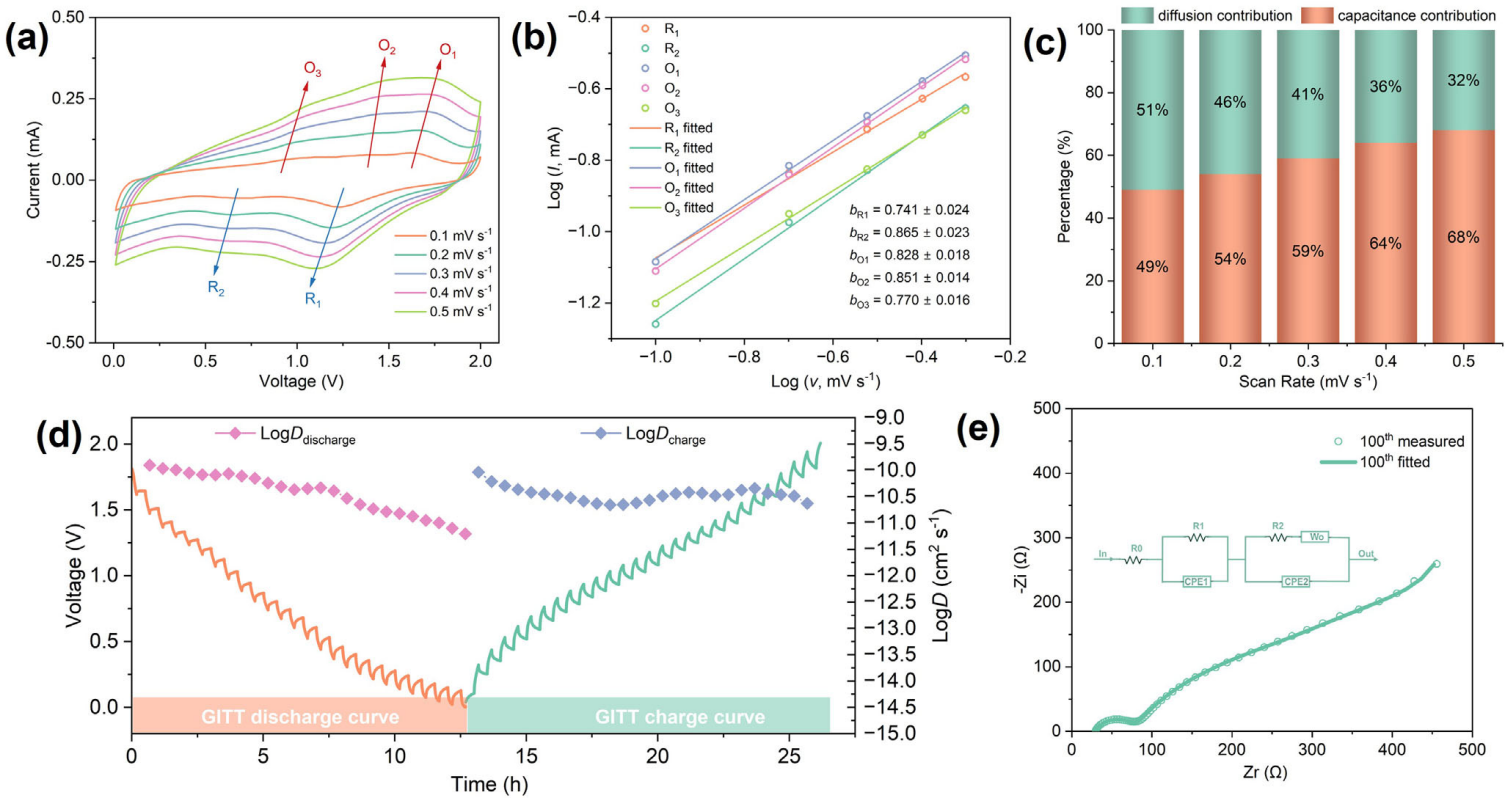
Electrochemical properties of the NbS_3_ electrode: (a) CV curves obtained at scan rates of 0.1–0.5 mV s^−1^. (b) Plots of the measured and fitted Log oxidation (violet, pink, and light green) and Log reduction (orange and cyan) peak currents against Log of scan rates *v*. (c) Histogram graph of the pseudo capacitative and diffusive proportions at various scan rates. (d) GITT curves (orange and cyan lines and bands) at 50 mA g^−1^ in the BMPyrr^+^‐containing electrolyte and corresponding diffusion coefficient plots (pink and violet scatter‐lines). (e) Nyquist plots (hollowed circles) and corresponding fitted curves (solid lines) of the Mg|APC‐BMPyrrCl|NbS_3_(+) cells after the 100th cycle. The equivalent circuits are shown as insets in the graph.

The charge storage behavior of the NbS_3_ electrode was further analysed using CV. CV curves collected at scan rates ranging from 0.1–0.5 mV s^−1^ (Figure 5a) were processed using a power law dependence (Equations S1 and S2). Linear fitting results (Figure 5b) yield *b* values for the reductive and oxidative peaks ranging from 0.7 to 0.9, suggesting that the charge storage mechanism involves a combination of pseudo capacitance and diffusion‐controlled processes. To quantify these components, Equation S3 was applied to process the current‐scan rate data, and the ratio of surface‐confined capacitive (*k*_1_) and bulk diffusion‐controlled (*k*_2_) processes was determined using the linear fitting of *i*/*v* ^1/2^ against 1/*v* ^1/2^. As shown in Figure 5c, the pseudo capacitance contributions were calculated to be 49%, 54%, 59%, 64%, and 68% at scan rates of 0.1, 0.2, 0.3, 0.4, and 0.5 mV s^−1^, respectively. These results indicate that at low scan rates, charge storage is governed by a combination of diffusion‐controlled and capacitive processes, whereas at high scan rates the capacitive contribution becomes dominant. The substantial capacitive component likely arises from multiple coupled factors supported by the mechanistic analysis, including BMPyrr^+^‐enabled channel opening and electrochemically induced morphological reconfiguration (i.e. nanosizing/fragmentation) [63, 64, 65, 66].

Galvanostatic intermittent titration technique (GITT) measurements were conducted to further assess magnesium ion diffusion within the NbS_3_ electrode. The diffusion coefficients (*D*) were calculated from the GITT (dis)charge data using Equation S4, as shown in Figure 5d. During discharge, the insertion diffusion coefficients remain relatively stable before decreasing to a minimum, corresponding to an increasing concentration of magnesium ions occupying the interlayer gaps. Conversely, the extraction diffusivities slightly decrease and then stabilise upon charging. Quantitatively, the *D* values during discharge and charge range from 6.22 × 10^−12^ to 1.26 × 10^−10^ cm^2^ s^−1^ and from 2.37 × 10^−11^ to 9.29 × 10^−11^ cm^2^ s^−1^, respectively. The average discharge diffusion coefficient was calculated to be 3.68 × 10^−11^ cm^2^ s^−1^, which is comparable to other state‐of‐art MIB cathode materials, such as expanded VS_2_ and TiS_2_ [18, 21]. Electrochemical impedance spectroscopy (EIS) was performed to further elucidate interfacial properties, charge transfer resistance, and ion diffusion kinetics in Mg|APC‐BMPyrrCl|NbS_3_ cells before and after 100 charge cycles. The Nyquist plots, shown in Figure S8 and Figure 5e, were fitted using a modified Randles circuit. In this model, R_0_ represents the bulk resistance contributed by the electrolyte and current collector. The high‐frequency semicircle, described by a parallel combination of resistance and a constant phase element (R_1_ + CPE_1_), corresponds to the solid‐electrolyte interface (SEI). The remaining elements of the circuit, including a second resistance, Warburg impedance (R_2_ + W_0_), and another constant phase element CPE_2_ in parallel, describe charge transfer and ion diffusion impedance in the cathode [67, 68]. Analysis of the fitted parameters (Table S3) reveals a substantial decrease in the interface resistance R_1_ from 17727 to 49 Ω after 100 cycles, likely due to the removal of passivating surface oxide layers and the establishment of a conductive SEI layer. Similarly, the charge transfer resistance, R_2_ significantly reduces from 10250 to 287 Ω, and the Warburg impedance, W_0_ declines from 785 Ω s^−0.5^ to 168 Ω s^−0.5^. These improvements suggest that after extended cycling, the pillaring effect of bulk BMPyrr^+^ cations between the pseudo‐layers enables efficient charge transfer and facilitates fast magnesium ion diffusion through the electrode particles, which significantly contributes to the enhanced electrochemical performance.

The combined electrochemical and mechanistic evidence supports a rapid first‐discharge activation of NbS_3_ by BMPyrr^+^ ions, rather than the multi‐cycle conditioning commonly reported for many layered sulfide cathodes. In pristine NbS_3_, the narrow vdW gap (*ca*. 0.29 nm) imposes high thermodynamic/kinetic barriers for ion intercalation and diffusion, leading to limited reversible magnesium storage. After BMPyrr^+^ introduction, however, the vdW gap expands to *ca*. 0.776 nm, weakening host‐carrier electrostatic interactions and facilitating reversible magnesium ion transport in widened channels. Consistently, persistent N 1s signals in both discharged and charged states further indicate that that BMPyrr^+^ insertion is established mainly in the first cycle and remains largely stable in subsequent cycling. This behavior contrasts with gradual activation in disulfide‐type hosts. A likely contributor is the unique sulfur chemistry of NbS_3_: carrier‐facing S_2_^2−^ moieties are redox‐active and less charge dense than S^2^, which can mitigate electrostatic interactions and lower barriers for initial BMPyrr^+^ entry and subsequent magnesium ion insertion, thereby accelerating early‐stage activation. In addition, structural topology may also contribute: (quasi‐)1D trichalcogenides consist of chain‐assembled pseudo‐layers that may provide more accessible insertion pathways and greater lattice compliance than densely stacked 2D disulfide layers, thereby facilitating faster BMPyrr^+^‐assisted channel opening. This interpretation is consistent with our prior BMPyrr^+^‐activated quasi‐1D TiS_3_ system [62], and with related behavior in S_2_^2−^ containing 1D VS_4_ [26], in contrast to the slower conditioning generally seen in S^2–^dominated 2D layered disulfide cathodes [22].

## 3 Conclusions

In conclusion, triclinic quasi‐1D pseudo‐layered NbS_3_ has been successfully synthesized and evaluated as a promising cathode material for MIBs. The key enabler of the activated magnesium ion storage in the NbS_3_ electrode is the interlayer expansion induced by the intercalation of electrolyte additive BMPyrr^+^. This expansion not only enhances magnesium ion diffusion kinetics, but, more critically, activates the inherent dual redox behavior of NbS_3_, specifically, the cationic Nb^4+^/Nb^3+^ and anionic S_2_^2–^/S^2–^ redox couples. This dual redox process facilitates multielectron transfer, as confirmed by *in operando* and ex situ PXRD, XPS, and XAS analyses. As a result, the expanded NbS_3_ electrode demonstrates a high reversible capacity of up to 200 mA h g^−1^ at 50 mA g^−1^, along with excellent cycling stability, far surpassing the negligible capacity of its non‐expanded counterpart. Additionally, multiple coupled factors, including BMPyrr**^+^**‐enabled channel opening and morphological reconfiguration (i.e., nanosizing and fragmentation) further contribute to substantial pseudo capacitance, enabling rapid (near‐)surface charge storage and efficient ion transport. This study underscores the promise of trichalcogenides as MIB cathodes and highlights interlayer expansion as a viable strategy to activate dual redox chemistry and achieve multielectron transfer for enhanced electrochemical performance. Future work will prioritize elucidating the decomposition pathways of NbS_3_ and mitigating sulfur loss under deep magnesium intercalation, while also establishing quantitative correlations between stage‐resolved micromechanical/microstructural evolution (e.g., particle‐size statistics) and pseudocapacitive contributions across different (dis)charge states. These efforts are essential for preserving the high capacities observed in early cycles and for realizing the full potential of trichalcogenide‐based MIBs.

## 4 Experimental Section

All experiments described below were conducted at room temperature (RT), unless explicitly stated otherwise.

### 4.1 Synthesis of NbS_3_

The NbS_3_ compound was synthesized via a physical vapor transport (PVT) method. In a typical synthesis, a slightly over‐stoichiometric molar ratio of niobium and sulfur powders (Nb:S = 1:3.06) was used. Specifically, 0.464 g Nb (99.8%, 325 mesh, Aldrich) and 0.490 g sulfur were thoroughly ground in an agate mortar under an argon atmosphere in a high‐purity Ar‐filled glove box. The homogenized mixture was then sealed in a quartz tube under static vacuum (ca. 10^−4^ mBar). The sealed tube was positioned horizontally in the center of a box furnace (BROTHER FURNACE, BR‐12N‐5) and heated using the following temperature profile: ramp at 200°C h^−1^ to 115°C and hold for 3 h, then ramp at 200°C h^−1^ to 550°C and maintain for 82 h [32]. Upon completion, the tube was cooled naturally to RT over ca. 4 h inside the furnace. The reacted tube was opened in the glove box using a molybdenum glass cutter, and the resulting product was collected and manually ground into fine particles before being stored under inert atmosphere. For electrode slurry preparation, 0.35 g of the as‐prepared NbS_3_ powder was mixed with 0.10 g of conductive carbon black. The mixture was homogenized via ball milling (AOT‐XQM‐0.4L planetary ball mill, stainless steel jar and five balls, ball‐to‐powder ratio 42:1) under Ar atmosphere at 400 rpm for 3 h with 2‐min forward and reverse rotations separated by 2‐min pauses.

### 4.2 Material Characterisation

Air‐stable as‐synthesized samples were characterized under ambient conditions, whereas air‐sensitive cycled samples were handled and measured under vacuum or inert atmosphere using customized sealed environments, such as flame‐sealed capillaries and air‐tight domed holders.

Comprehensive characterisation was performed using powder X‐ray diffraction (PXRD), Raman spectroscopy, X‐ray photoelectron spectroscopy (XPS), X‐ray absorption spectroscopy (XAS), scanning electron microscopy with energy‐dispersive X‐ray spectroscopy (SEM‐EDS), and transmission electron microscopy (TEM). Structural evolution of the NbS_3_ electrode during electrochemical cycling was investigated using a bespoke *in operando* PXRD cell constructed in‐house. For initial structural analysis, PXRD patterns were collected in flat plate reflection mode (Bragg‐Brentano geometry) over a 2*θ* range of 5°–70°, with a step size of 0.0175° and a scan rate of 0.083° s^−1^, using a Rigaku MiniFlex diffractometer equipped with an unmonochromated Cu K_α_ radiation (40 kV, 40 mA). *In operando* and ex situ PXRD patterns were recorded using Bragg‐Brentano (5°–60°, step size 0.026°, scan rate 0.067° s^−1^) using a PANalytical Empyrean diffractometer with an unmonochromated Cu K_α_ X‐ray source at 45 kV and 40 mA. Raman spectra were collected using a LabRAM HR spectrometer equipped with a 532 nm green laser. XPS analyses were conducted to study the oxidation states of Nb and S using Nexsa X‐ray photoelectron spectrometers with an Al K_α_ X‐ray source. High‐resolution XPS spectra were fitted using dual Gaussian‐Lorentzian functions according to the procedure described by Conny and Powell [69, 70]. XAS measurements at the Nb L‐edge and S K‐edge were performed at the BL2A beamline of the UVSOR Synchrotron Facility (Institute for Molecular Science, Japan). Spectra were acquired in total fluorescence yield mode and calibrated using the S K‐edge spectrum of Li_2_S. The morphologies and spatially resolved elemental composition of NbS_3_ samples were characterized using a combination of SEM (TESCAN CLARA, 15 kV) equipped with an Oxford Instrument UltimMax 65 EDS detector, and TEM (FEI Tecnai G2 F30 Microscope, 200 kV).

### 4.3 Electrochemical Measurements

The electrochemical performance of NbS_3_ electrodes was evaluated using CR2032‐type coin cells. Magnesium foil discs (15 mm diameter, 0.2 mm thickness, 99.5%, Huabei Magnesium Processing Plant) served as the anodes. A cathode slurry was prepared by mixing 0.07 g of active material, 0.02 g of conductive carbon (carbon black, 99%, Alfa Aesar), and 0.01 g of polyvinyldifluorine (PVDF, 98%, average molecular weight ∼534 000, Sigma–Aldrich) in ca. 0.5 mL of N‐methyl pyrrolidone (NMP, anhydrous, 99.5%, Sigma–Aldrich). The resulting slurry was cast onto Ni foam chips (99.8%, 12 mm diameter, 1 mm thickness, areal density 280–420 g m^−2^, Saibo Electrochemistry) and dried under static vacuum (0.1 mbar) in an oven at 60°C overnight. The unmodified electrolyte, 0.4 m “All‐phenyl complex” (APC), was prepared by the dropwise addition of 4.0 mL phenyl magnesium chloride (PhMgCl, 2.0 m in tetrahydrofuran (THF), Sigma–Aldrich) into a solution of 0.534 g aluminim chloride (AlCl_3_, ultra dry, 99.99%, Thermo Fisher Scientific) in 6 mL THF (≥ 99.9%, anhydrous, inhibitor‐free, Sigma–Aldrich) under magnetic stirring. To prepare the additive‐containing electrolyte, 0.089 g of 1‐butyl‐1‐methylpyrrolidinium chloride (BMPyrrCl, 99%, Aladdin) was added to 2 mL of APC.

Galvanostatic discharge–charge cycling and galvanostatic intermittent titration technique (GITT) measurements were conducted using a LAND CT2001A battery tester over a voltage window of 0.01–2.0 V. For GITT, the cell was pulsed with a current density of 50 mA g^−1^ for 600 s, followed by a relaxation period (no current applied) of 1200 s. These current/pause sequences were repeated until the lower and upper voltage limits (0.01 V and 2.0 V, respectively) were reached. The calculation of diffusion coefficients from the GITT data is described in Equation S4 and illustrated in Figure S7. Cyclic voltammetry (CV) measurements were performed at scan rates of 0.1–0.5 mV s^−1^, beginning from open‐circuit voltage to 0.01 V, followed by a reverse scan to 2.0 V). Electrochemical impedance spectroscopy (EIS) was conducted over a frequency range 100 kHz to 0.01 Hz with a 10‐mV amplitude at RT. The resulting Nyquist plots were fitted using the AfterMath software [71]. Both CV and EIS were performed on a PalmSens4 potentiostat.

## Funding

University of Glasgow and China Scholarship Council PhD studentship; the IMS Program (Japan) with grant code 24IMS6007; Grant‐in‐Aid for “2019 Initiative for Realizing Diversity in the Research Environment”; Adopting Sustainable Partnerships for Innovative Research Ecosystem (ASPIRE), Grant Number JPMJAP2419.

## Conflicts of Interest

The authors declare no conflicts of interest.

## Supporting information




**Supporting File 1**: advs74690‐sup‐0001‐SuppMat.docx.

**Supporting File 2**: advs74690‐sup‐0002‐Figure S1.pptx.

**Supporting File 3**: advs74690‐sup‐0003‐Figure S2.pptx.

**Supporting File 4**: advs74690‐sup‐0004‐Figure S3.pptx.

**Supporting File 5**: advs74690‐sup‐0005‐Figure S4.pptx.

**Supporting File 6**: advs74690‐sup‐0006‐Figure S5.pptx.

**Supporting File 7**: advs74690‐sup‐0007‐Figure S6.pptx.

**Supporting File 8**: advs74690‐sup‐0008‐Figure S7.pptx.

**Supporting File 9**: advs74690‐sup‐0009‐Figure S8.pptx.

## Data Availability

The data that support the findings of this study are available from the corresponding author upon reasonable request.
