# Supplementary material for: In Situ Electrochemical Activation of Pseudo‐Layered NbS3 via Interlayer Expansion and Dual Redox for High Mg‐Ion Storage
Source: Adv Sci (Weinh). 2026 Mar 7;13(27):e74690. doi: 10.1002/advs.74690 (PMC13170196; doi:10.1002/advs.74690)
Supplement: Supplementary file 1 — Supporting File 1: advs74690‐sup‐0001‐SuppMat.docx. [file ADVS-13-e74690-s001.docx]

Supporting Information

*In Situ* Electrochemical Activation of Pseudo-Layered NbS_3_ *via* Interlayer Expansion and Dual Redox for High Mg-Ion Storage

Pengcheng Jing, Atsushi Inoishi, Chengcheng Zhao, Eiichi Kobayashi, Peng Ren, Isaac Abrahams and Duncan H. Gregory *

* Corresponding author

Pengcheng Jing

WestCHEM, School of Chemistry, Joseph Black Building, University of Glasgow, Glasgow, UK, G12 8QQ
Tianmushan Laboratory, Yuhang District, Hangzhou, China, 311115

Chengcheng Zhao, Duncan H. Gregory

WestCHEM, School of Chemistry, Joseph Black Building, University of Glasgow, Glasgow, UK, G12 8QQ
E-mail:  [Duncan.Gregory@glasgow.ac.uk](mailto:Duncan.Gregory@glasgow.ac.uk)

Atsushi Inoishi
Institute for Materials Chemistry and Engineering, Kyushu University, Kasuga-koen 6-1, Kasuga, Fukuoka, Japan, 816-8580.

Eiichi Kobayashi

Kyushu Synchrotron Light Research Center, 8-7 Yayoigaoka, Tosu, Saga, Japan, 841-0005.

Peng Ren, Isaac Abrahams

Department of Chemistry, Queen Mary University of London, Mile End Road, London, UK, E1 4NS.


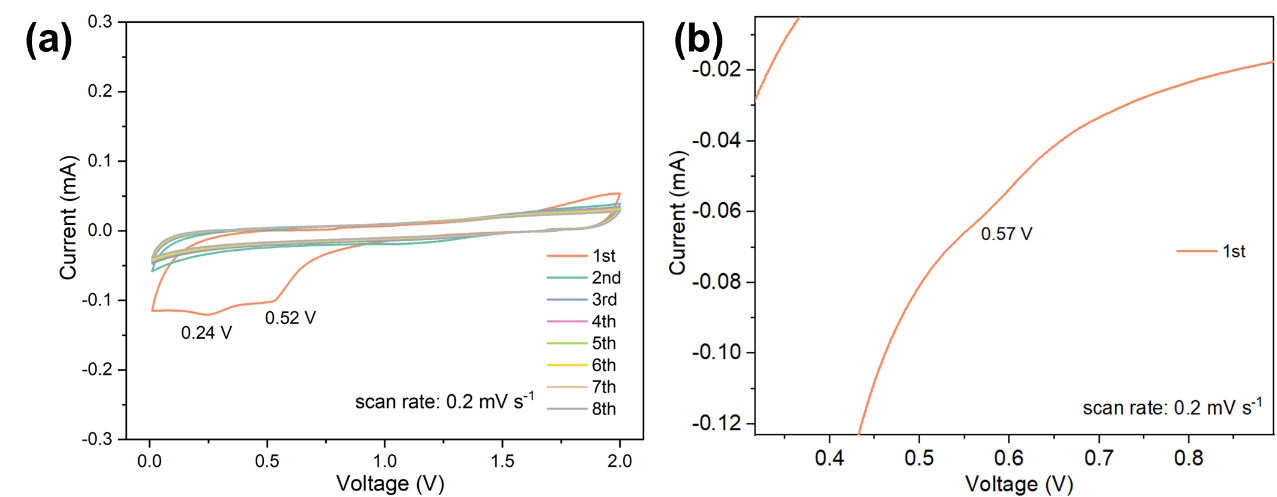


**Figure S1.** (a) Initial 8 cycles of CV curves of the NbS_3_ electrode in the *unmodified* APC electrolyte at a scan rate of 0.2 mV s^-1^. (b) Zoomed region of the first CV curve in *modified* APC electrolyte.


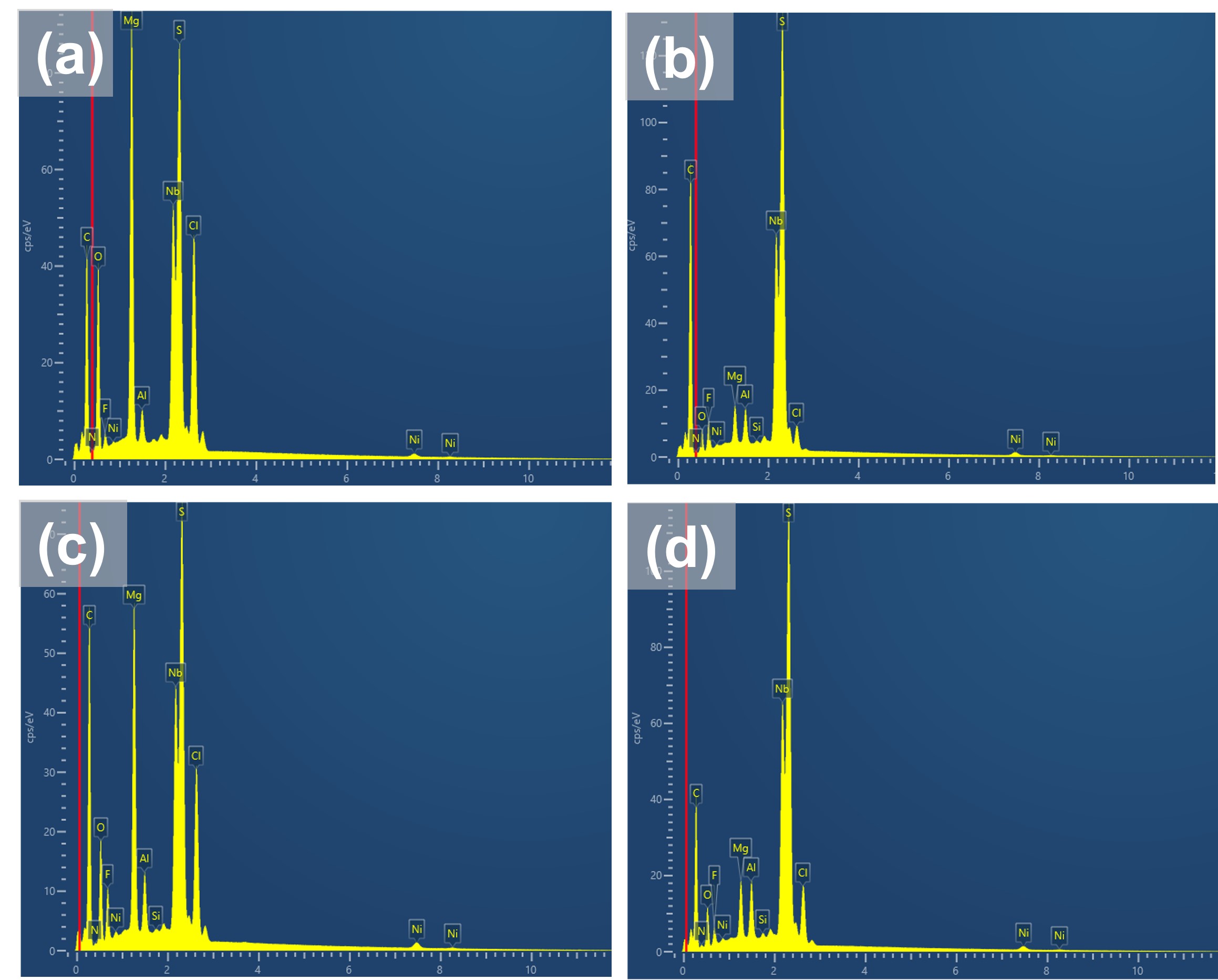


**Figure S2.** EDS spectra from elemental maps of the NbS_3_ electrodes at: (a) 1D0.01, (b) 1C2.0V, (c) 2D0.01V, and (d) 2C2.0V (dis)charge states.

**Table S1.** As-detected atomic composition of the NbS_3_ electrodes as-derived from Figure S2 and processed atomic ratios (shadowed area) of different elements at various (dis)charge states.

| **As Detected/ At.%** | | | | | | **Atomic Ratios** | | | | | |
| --- | --- | --- | --- | --- | --- | --- | --- | --- | --- | --- | --- |
| **State** | **Mg%** | **Cl/%** | **Al/%** | **Nb/%** | **S/%** | **Mg/Cl** | **Cl/Al** | **Mg/Nb** | **Cl/ Nb** | **Al/ Nb** | **S/ Nb** |
| **1D0.01V** | 6.70 | 4.20 | 0.50 | 2.90 | 5.50 | 1.60 | 8.40 | 2.31 | 1.45 | 0.17 | 1.90 |
| **1C2.0V** | 0.70 | 0.60 | 0.60 | 3.00 | 7.20 | 1.17 | 1.00 | 0.23 | 0.20 | 0.20 | 2.40 |
| **2D0.01V** | 4.30 | 2.80 | 0.70 | 2.60 | 5.00 | 1.54 | 4.00 | 1.65 | 1.08 | 0.27 | 1.92 |
| **2C2.0V** | 1.40 | 1.90 | 1.20 | 4.40 | 9.20 | 0.74 | 1.58 | 0.32 | 0.43 | 0.27 | 2.09 |


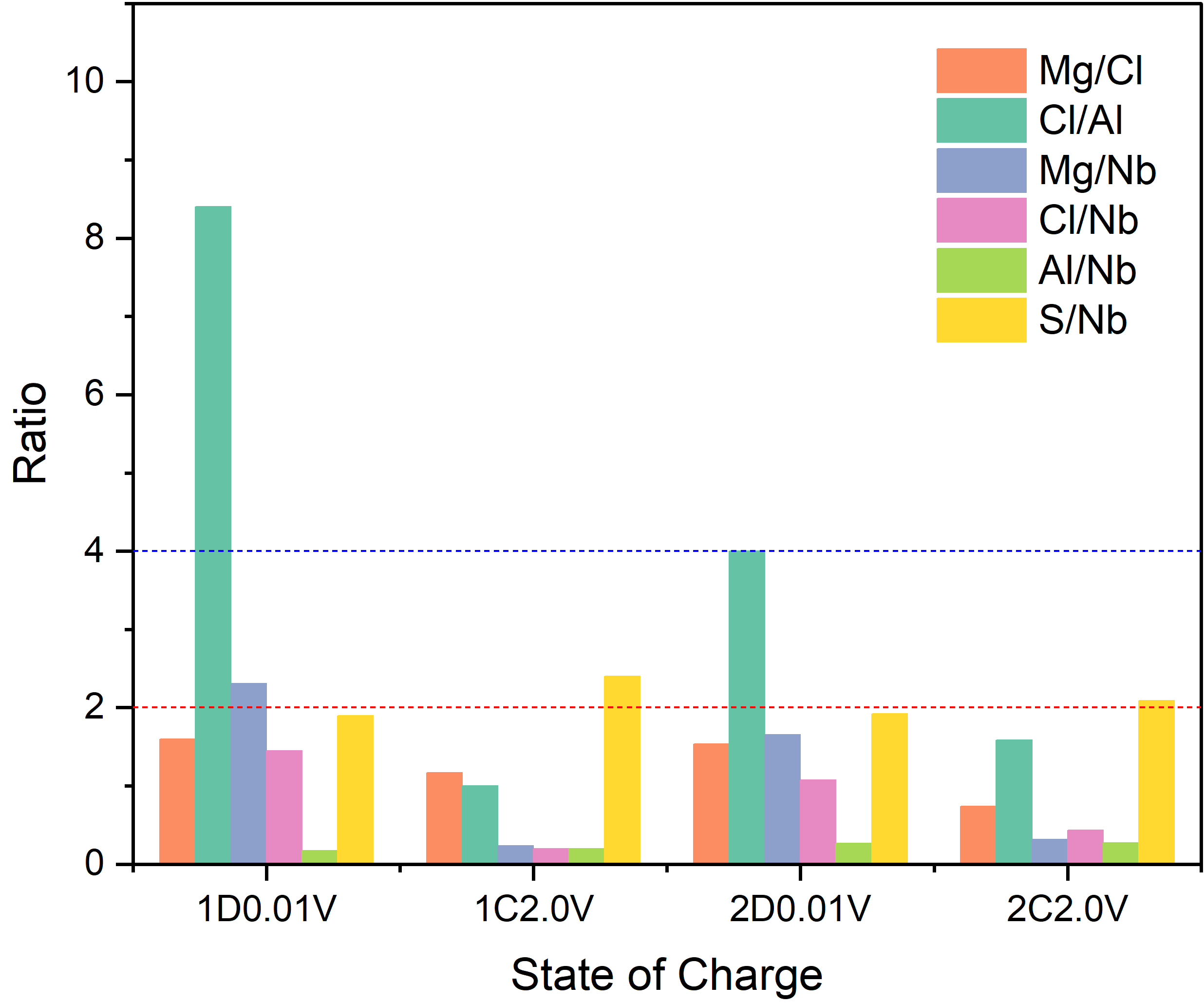


**Figure S3.** Histogram graph of atomic ratios of different elements of the electrode material at different states of charge.


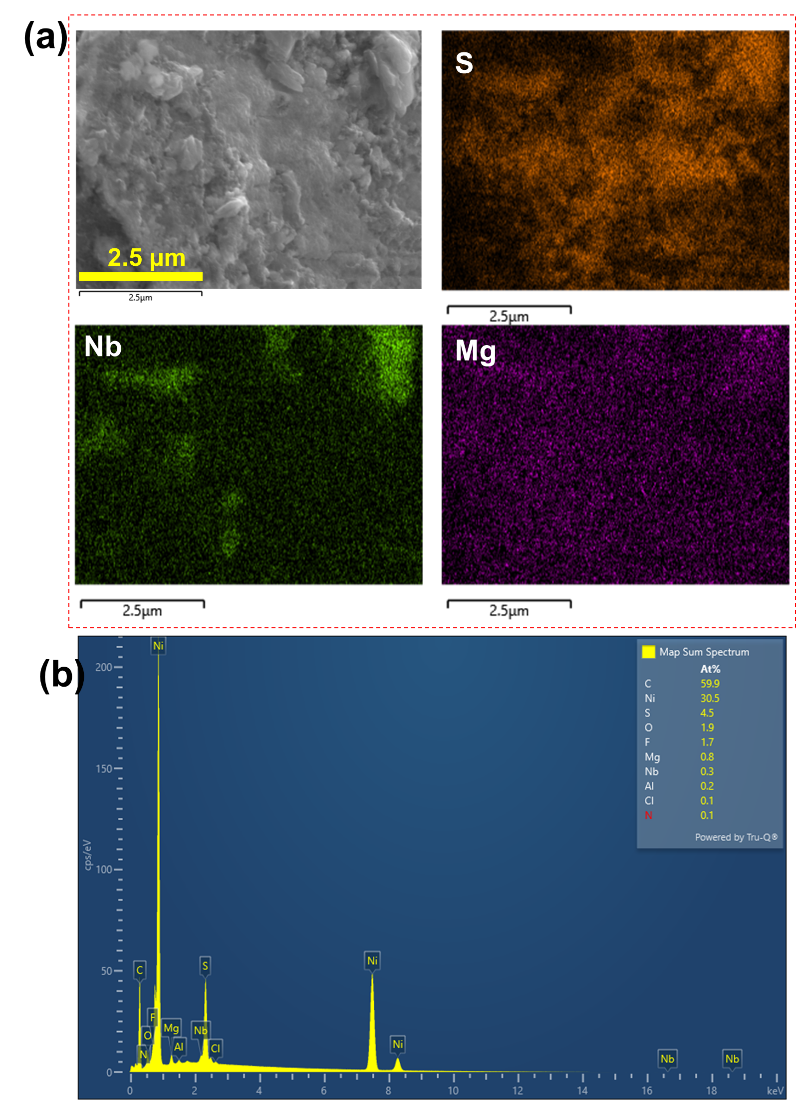


**Figure S4.** (a) SEM-EDS maps of S, Nb, and Mg and (b) EDS spectrum in the sulfur-rich NbS_3_ electrode region.


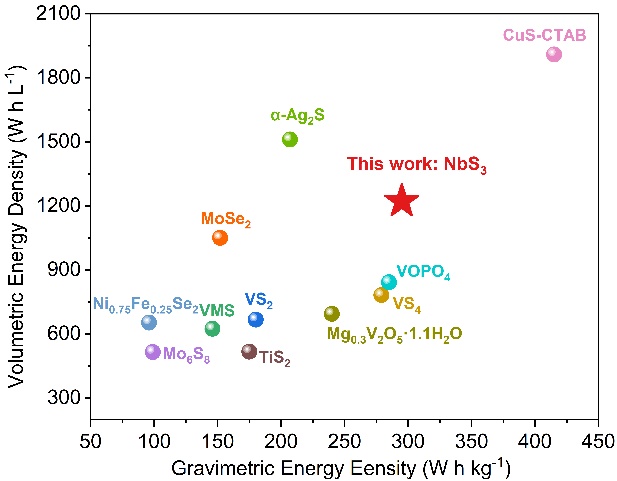


**Figure S5.** Comparison of achievable gravimetric and volumetric energy densities between NbS_3_ (this work) and representative reported MIB cathode materials. Data presented in the graph were processed from Table S2.

**Table S2.** Comparison of achievable gravimetric and volumetric energy densities between NbS_3_ (this work) and representative reported MIB cathode materials.

| **Cathode** | **Electrolyte** | **Current Density**  **(mA g^-1^)** | **Reversible Capacity**  **(mA h g^-1^)** | | **Gravimetric Energy Density**  **(W h Kg^−1^)** | **Volumetric Energy Density**  **(W h L^−1^)** | **Refs** |
| --- | --- | --- | --- | --- | --- | --- | --- |
| **CuS-CTAB** | Mg[B(hfip)_4_]_2_/DME | 50 | 477 | 415 | | 1909 | [1] |
| **VS_2_** | APC | 100 | 234 | 180 | | 667 | [2] |
| **VMS** | APC-BMPyrrCl | 100 | 211 | 146 | | 624 | [3] |
| **Mo_6_S_8_** | BEC | \ | 90 | 99 | | 515 | [4] |
| **VS_4_** | APC | 50 | 274 | 279 | | 782 | [5] |
| **VOPO_4_** | APC | 50 | 300 | 285 | | 842 | [6] |
| **TiS_2_** | APC-BMPyrrCl | 48 | 250 | 175 | | 517 | [7] |
| **Mg_0.3_V_2_O_5_·1.1H_2_O** | APC | 100 | 160 | 240 | | 694 | [8] |
| **MoSe_2_** | MgCl_2_-Mg(TFSI)_2_ | 50 | 282 | 152 | | 1050 | [9] |
| **Ni_0.75_Fe_0.25_Se_2_** | APC | 50 | 120 | 96 | | 653 | [10] |
| **α-Ag_2_S** | APC | 50 | 159 | 207 | | 1511 | [11] |
| **NbS_3_** | APC | 50 | 343 | 295 | | 1221 | This work |

The calculation basis and data sources are provided below:

*E*_g_ = *Q* × *V*_mid_, *E*_v_ = *E*_g_ × *ñ*,

Where *Q* is achievable reversible capacity, *V*_mid_ is median discharge voltage, and *ñ* is the cathode density used for volumetric conversion.


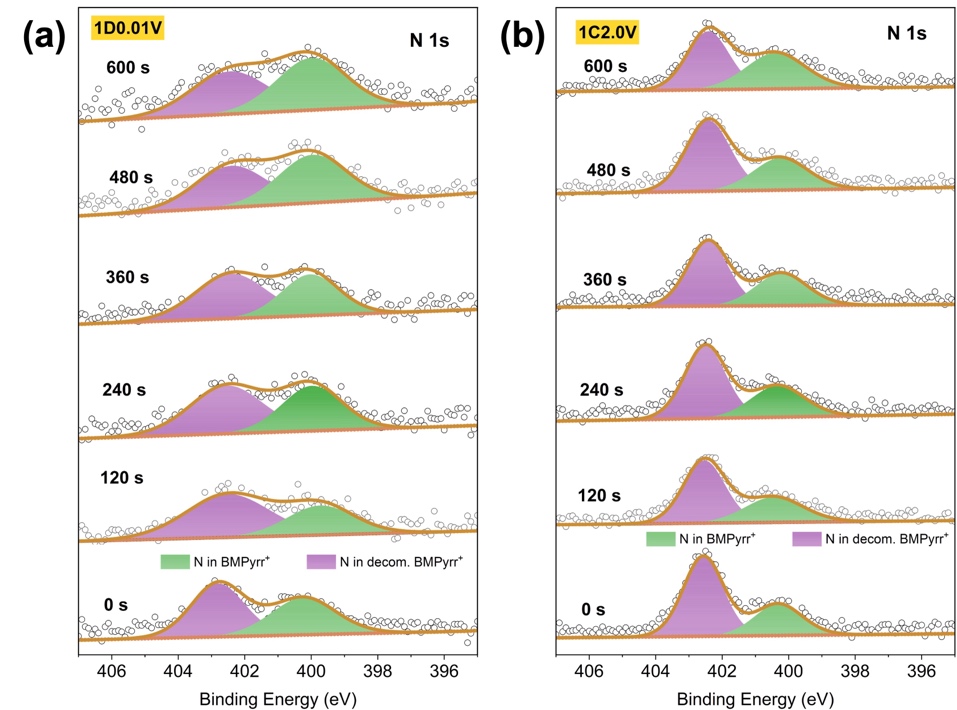


**Figure S6.** High-resolution N 1s spectra of fully discharged and charged NbS_3_ electrodes after sputtering for various durations using an Ar^+^ beam.

The CV peak current follows a power-law dependence on scan rate (Equation S1):[12]

$i=av^{b}$ Equation S1

where $i$is the current (mA), $v$is the scan rate (mV s^-1^), and $a$and $b$are fitting constants. A $b$-value of 0.5 indicates a diffusion-limited process ($i\propto v^{1/2}$), whereas $b=1.0$corresponds to a surface-controlled capacitive process ($i\propto v$). For mixed kinetics, $b$lies between 0.5 and 1.0. The $b$-value is obtained from the slope of the $\log(i)$-$\log(v)$ plot (Equation S2):

$log(i)=log(a)+blog(v)$ Equation S2

To further separate current contributions at a given potential, the total current can be expressed as Equation S3:[13]

$i=k_{1}v+k_{2}v^{1/2}\mathrm{or}\frac{i}{v^{1/2}}=k_{1}v^{1/2}+k_{2}$ Equation S3

where $k_{1}v$represents the capacitive (surface-controlled) contribution and $k_{2}v^{1/2}$represents the diffusion-controlled contribution. Thus, linear fitting of $i/v^{1/2}$versus $v^{1/2}$enables quantitative estimation of both components.

The ion diffusion coefficient ($D$) was estimated from GITT measurements using Fick’s second law (Equation S4):[14]

$D=\frac{4}{}\left( \frac{m_{B}V_{M}}{M_{B}S} \right)^{2}\left( \frac{Es}{E\tau} \right)^{2}$ Equation S4

where $\tau$is the current-pulse duration (s), $m_{B}$is the mass of active material (g), $V_{M}$is the molar volume (cm^3^ mol^-1^), $M_{B}$is the molar mass (g mol^-1^), and $S$is the electrode area (cm^2^). $\Delta E_{\tau}$is the transient voltage change during the pulse after IR-drop correction, [3, 15] and $\Delta E_{s}$is the steady-state potential change between two equilibrium states before and after the pulse. Both values were extracted from the GITT profiles (Figure 5d).


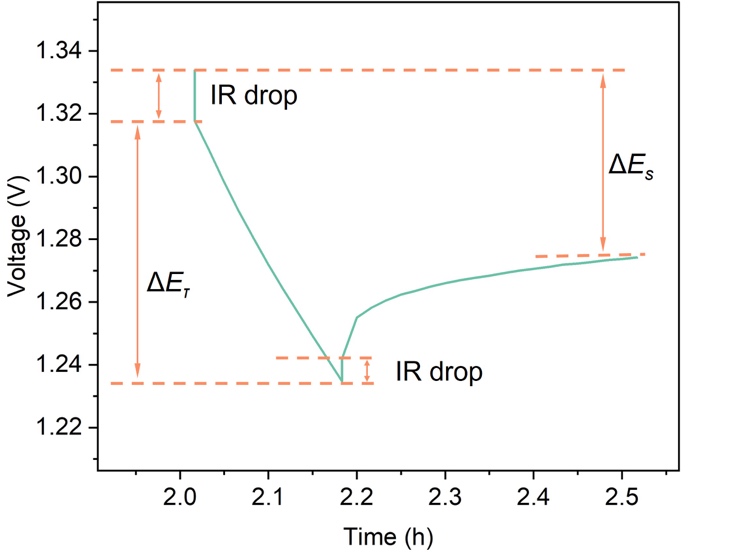


**Figure S7.** One pair of current pulse and relaxation data taken from the GITT curve of the NbS_3_ electrode shown in Figure 5d, illustrating the measurements of Δ*E*_τ_, Δ*E*_s_*,* and the *IR* drop, respectively.


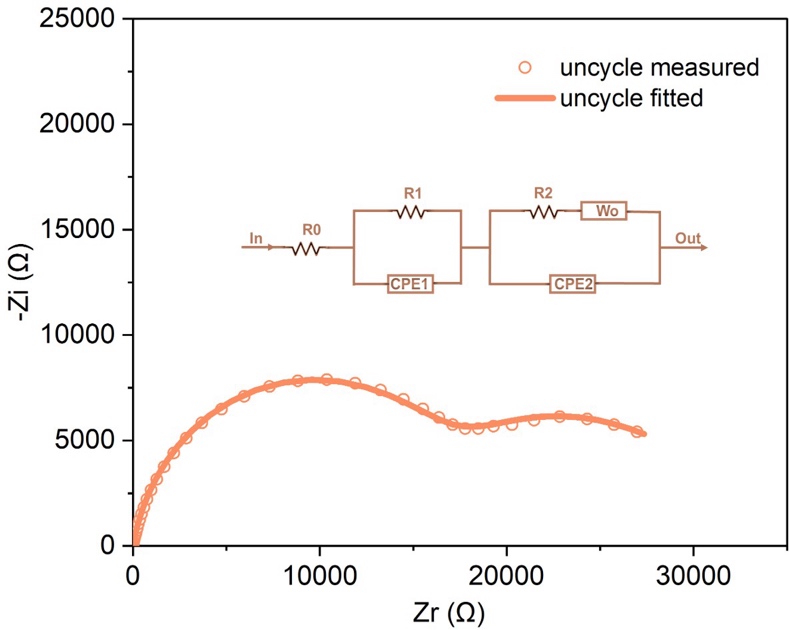


**Figure S8.** Nyquist plots (open circles) and corresponding fitted curves (solid lines) of the Mg|APC-BMPyrrCl|NbS_3_(+) cells before cycle. The equivalent circuits are shown as insets in the graph.

**Table S3.** Values of parameters derived from the equivalent circuit model of the Mg|APC-BMPyrrCl|NbS_3_ cells at different two selected states of charge.

|  |  | **Q_1_(sᵅ Ω^-1^)** | **Q_2_(sᵅ Ω^-1^)** | **R_0_(Ω)** | **R_1_(Ω)** | **R_2_(Ω)** | **W_0_(Ω s^-0.5^)** |
| --- | --- | --- | --- | --- | --- | --- | --- |
| **Before cycle** | value | 1.27E-05 | 3.80E-04 | 7.25 | 17727.40 | 10250.20 | 784.76 |
|  | error | 3.47E-11 | 5.12E-06 | 0.12 | 272.33 | 154.55 | 13.28 |
| **100^th^ charge** | value | 3.12E-05 | 1.63E-03 | 29.37 | 49.15 | 286.94 | 168.46 |
|  | error | 9.35E-09 | 1.37E-04 | 2.64 | 4.06 | 24.61 | 14.71 |

**References**

[1] Y. Shen, Y. Wang, Y. Miao, M. Yang, X. Zhao, X. Shen, *Advanced Materials* **2020**, *32* (4), 1905524, <https://doi.org/https://doi.org/10.1002/adma.201905524>.

[2] P. Jing, H. Lu, W. Yang, Y. Cao, *Electrochimica Acta* **2020**, *330*, 135263, <https://doi.org/https://doi.org/10.1016/j.electacta.2019.135263>.

[3] P. Jing, S. Stevenson, H. Lu, P. Ren, I. Abrahams, D. H. Gregory, *ACS Applied Materials & Interfaces* **2023**, *15* (44), 51036, <https://doi.org/10.1021/acsami.3c10287>.

[4] D. Aurbach, Z. Lu, A. Schechter, Y. Gofer, H. Gizbar, R. Turgeman, Y. Cohen, M. Moshkovich, E. Levi, *Nature* **2000**, *407* (6805), 724, <https://doi.org/10.1038/35037553>.

[5] Y. Man, A. Li, H. Tang, J. Sun, Y. Fei, Y. Du, X. Zhou, *Science China Chemistry* **2024**, *67* (9), 3153, <https://doi.org/10.1007/s11426-024-2195-2>.

[6] L. Zhou, Q. Liu, Z. Zhang, K. Zhang, F. Xiong, S. Tan, Q. An, Y.-M. Kang, Z. Zhou, L. Mai, *Advanced Materials* **2018**, *30* (32), 1801984, <https://doi.org/https://doi.org/10.1002/adma.201801984>.

[7] H. D. Yoo, Y. Liang, H. Dong, J. Lin, H. Wang, Y. Liu, L. Ma, T. Wu, Y. Li, Q. Ru, Y. Jing, Q. An, W. Zhou, J. Guo, J. Lu, S. T. Pantelides, X. Qian, Y. Yao, *Nature Communications* **2017**, *8* (1), 339, <https://doi.org/10.1038/s41467-017-00431-9>.

[8] Y. Xu, X. Deng, Q. Li, G. Zhang, F. Xiong, S. Tan, Q. Wei, J. Lu, J. Li, Q. An, L. Mai, *Chem* **2019**, *5* (5), 1194, <https://doi.org/https://doi.org/10.1016/j.chempr.2019.02.014>.

[9] D. Chen, X. Ren, T. Li, Z. Chen, Y. Cao, F. Xu, *ENERGY & ENVIRONMENTAL MATERIALS* **2023**, *6* (6), e12486, <https://doi.org/https://doi.org/10.1002/eem2.12486>.

[10] L. Zhou, F. Xiong, S. Tan, Q. An, Z. Wang, W. Yang, Z. Tao, Y. Yao, J. Chen, L. Mai, *Nano Energy* **2018**, *54*, 360, <https://doi.org/https://doi.org/10.1016/j.nanoen.2018.10.033>.

[11] Z. Chen, Z. Zhang, A. Du, Y. Zhang, M. Men, G. Li, G. Cui, *Chemical Communications* **2019**, *55* (30), 4431, <https://doi.org/10.1039/C9CC01638D>.

[12] W. Ren, H. Zhang, C. Guan, C. Cheng, *Adv. Funct. Mater.* **2017**, *27* (32), 1702116, <https://doi.org/https://doi.org/10.1002/adfm.201702116>.

[13] J. Wang, J. Polleux, J. Lim, B. Dunn, *The Journal of Physical Chemistry C* **2007**, *111* (40), 14925, <https://doi.org/10.1021/jp074464w>.

[14] C. J. Wen, B. A. Boukamp, R. A. Huggins, W. Weppner, *Journal of The Electrochemical Society* **1979**, *126* (12), 2258, <https://doi.org/10.1149/1.2128939>.

[15] K. W. Knehr, S. Biswas, D. A. Steingart, *Journal of The Electrochemical Society* **2017**, *164* (13), A3101, <https://doi.org/10.1149/2.0821713jes>.
