# Supplementary figures and images for: In Situ Electrochemical Activation of Pseudo‐Layered NbS3 via Interlayer Expansion and Dual Redox for High Mg‐Ion Storage
Source: Adv Sci (Weinh). 2026 Mar 7;13(27):e74690. doi: 10.1002/advs.74690 (PMC13170196; doi:10.1002/advs.74690)

## Slide 1
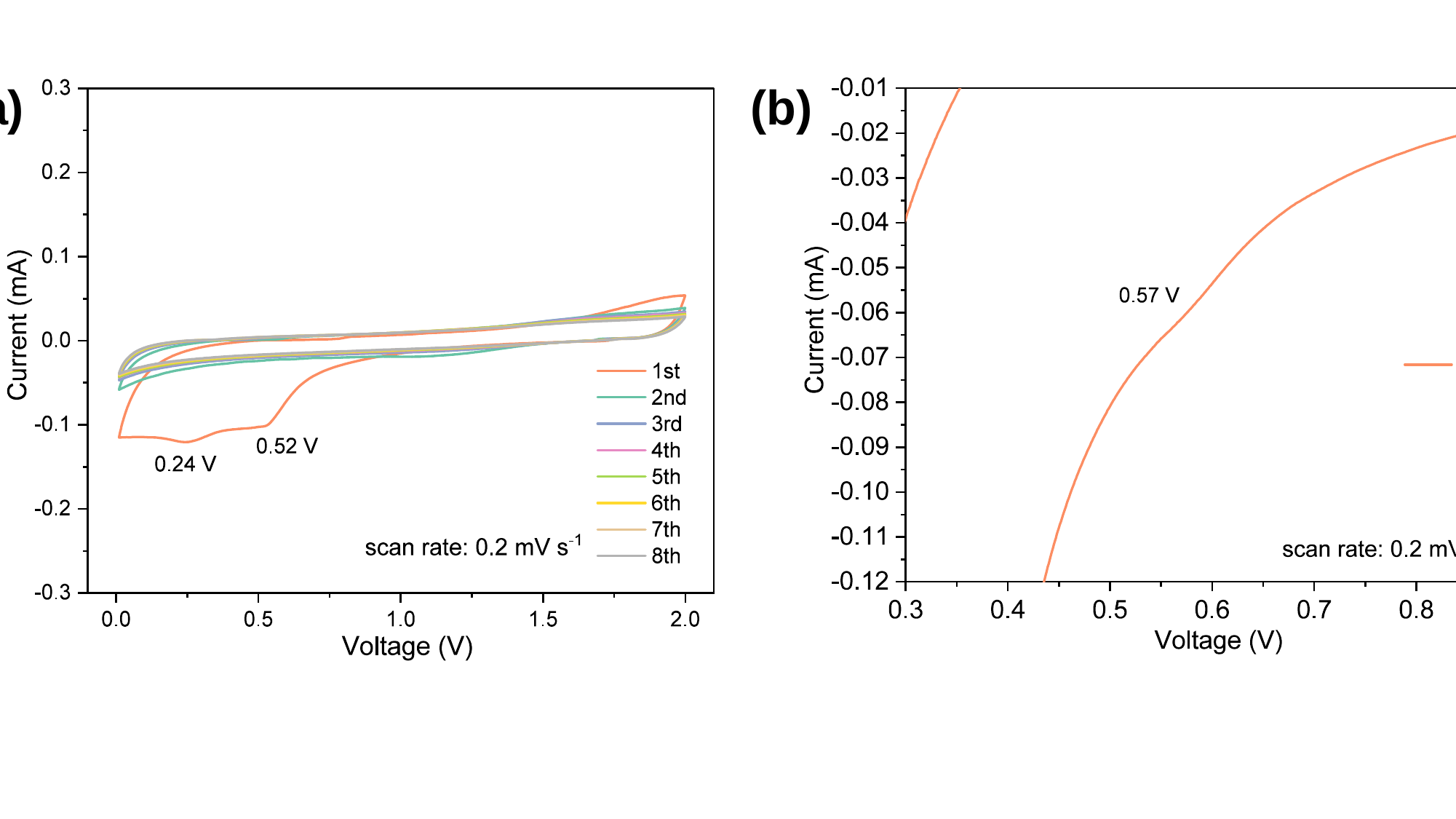

(b)
(a)

Supplement: Supplementary file 2 — Supporting File 2: advs74690‐sup‐0002‐Figure S1.pptx. [file ADVS-13-e74690-s004.pptx]

## Slide 1
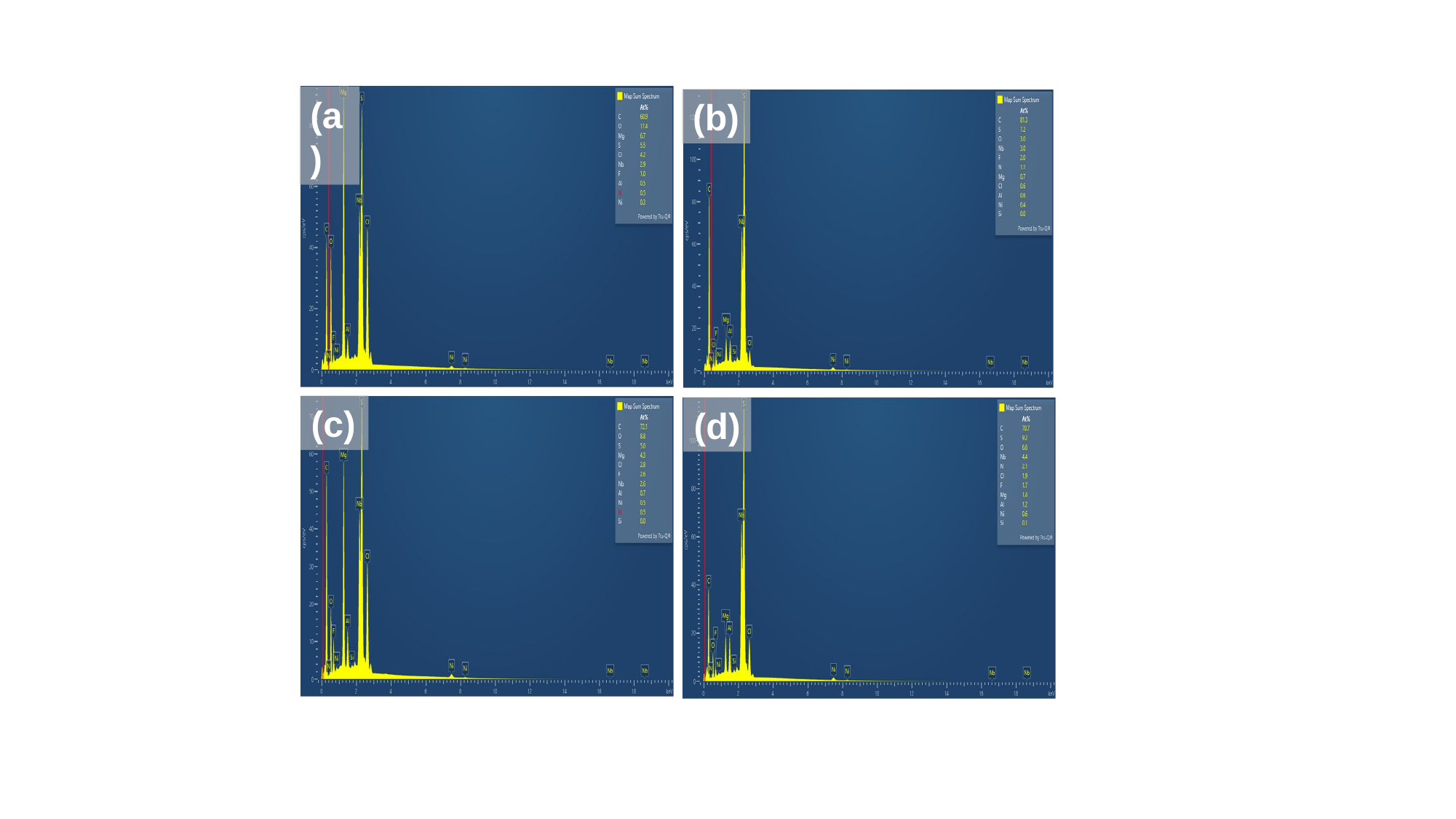

(a)
(b)
(c)
(d)

Supplement: Supplementary file 3 — Supporting File 3: advs74690‐sup‐0003‐Figure S2.pptx. [file ADVS-13-e74690-s005.pptx]

## Slide 1
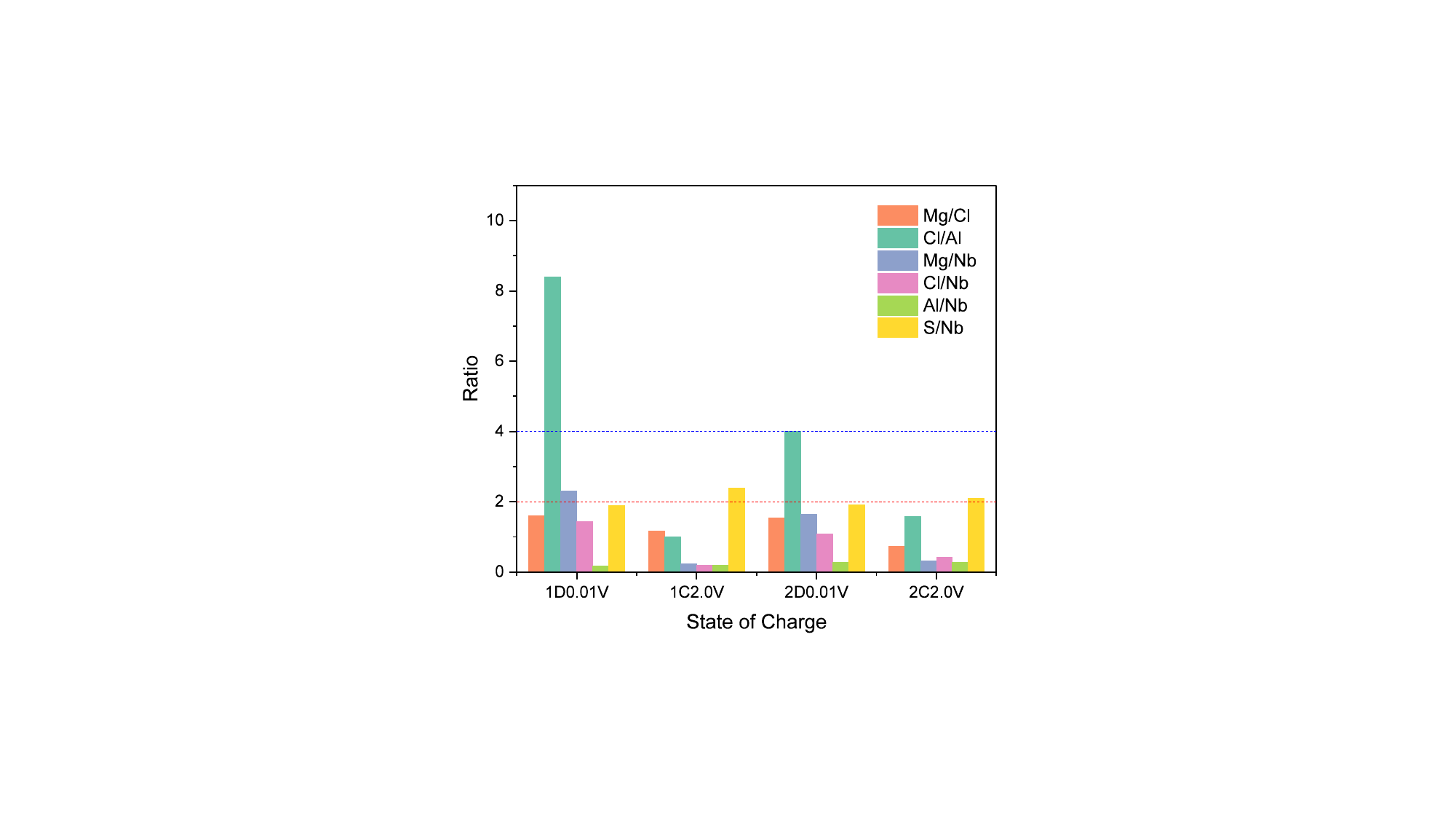

Supplement: Supplementary file 4 — Supporting File 4: advs74690‐sup‐0004‐Figure S3.pptx. [file ADVS-13-e74690-s002.pptx]

## Slide 1
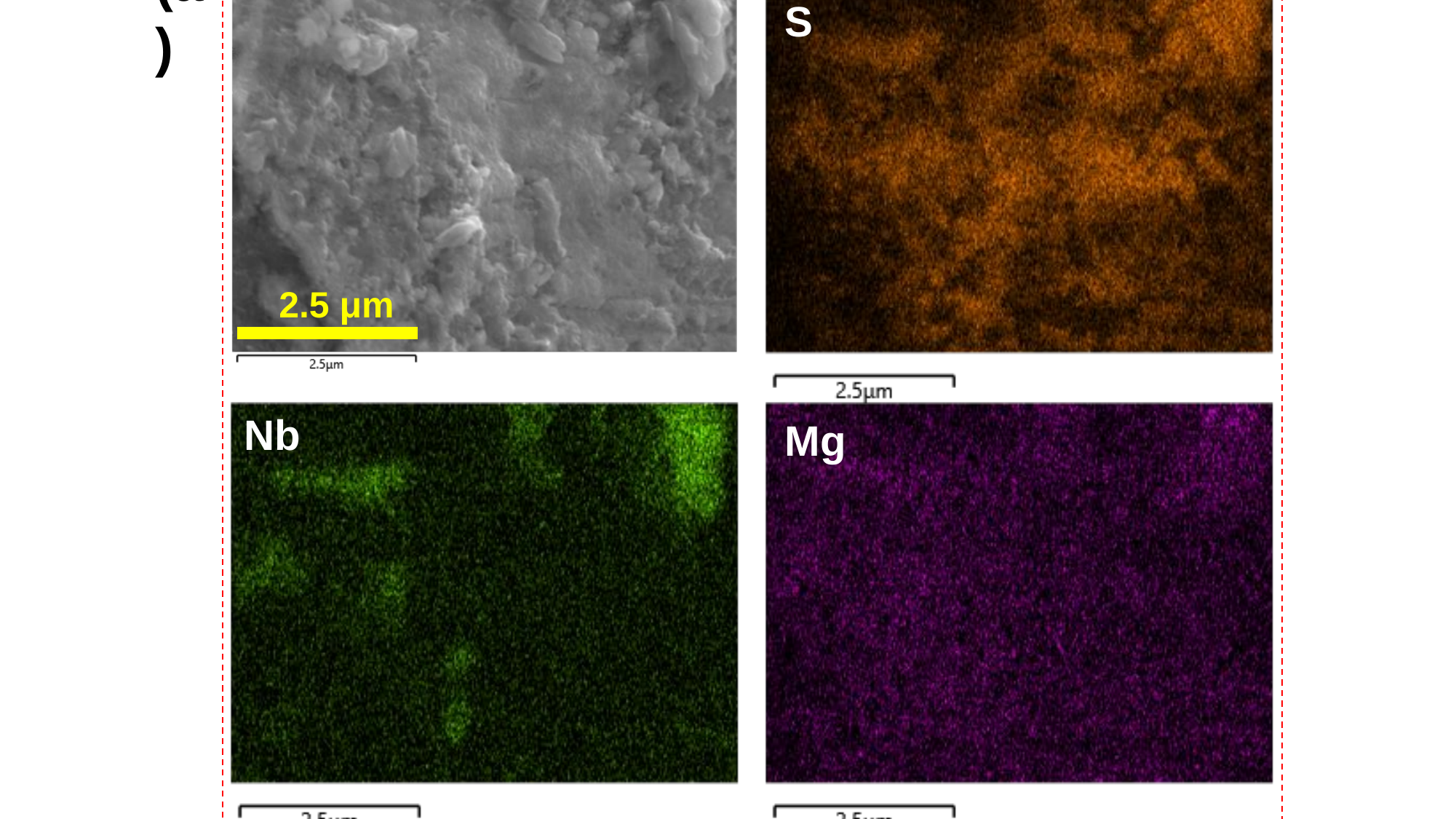

(a)
S
2.5 μm
Nb
Mg
(b)

Supplement: Supplementary file 5 — Supporting File 5: advs74690‐sup‐0005‐Figure S4.pptx. [file ADVS-13-e74690-s007.pptx]

## Slide 1
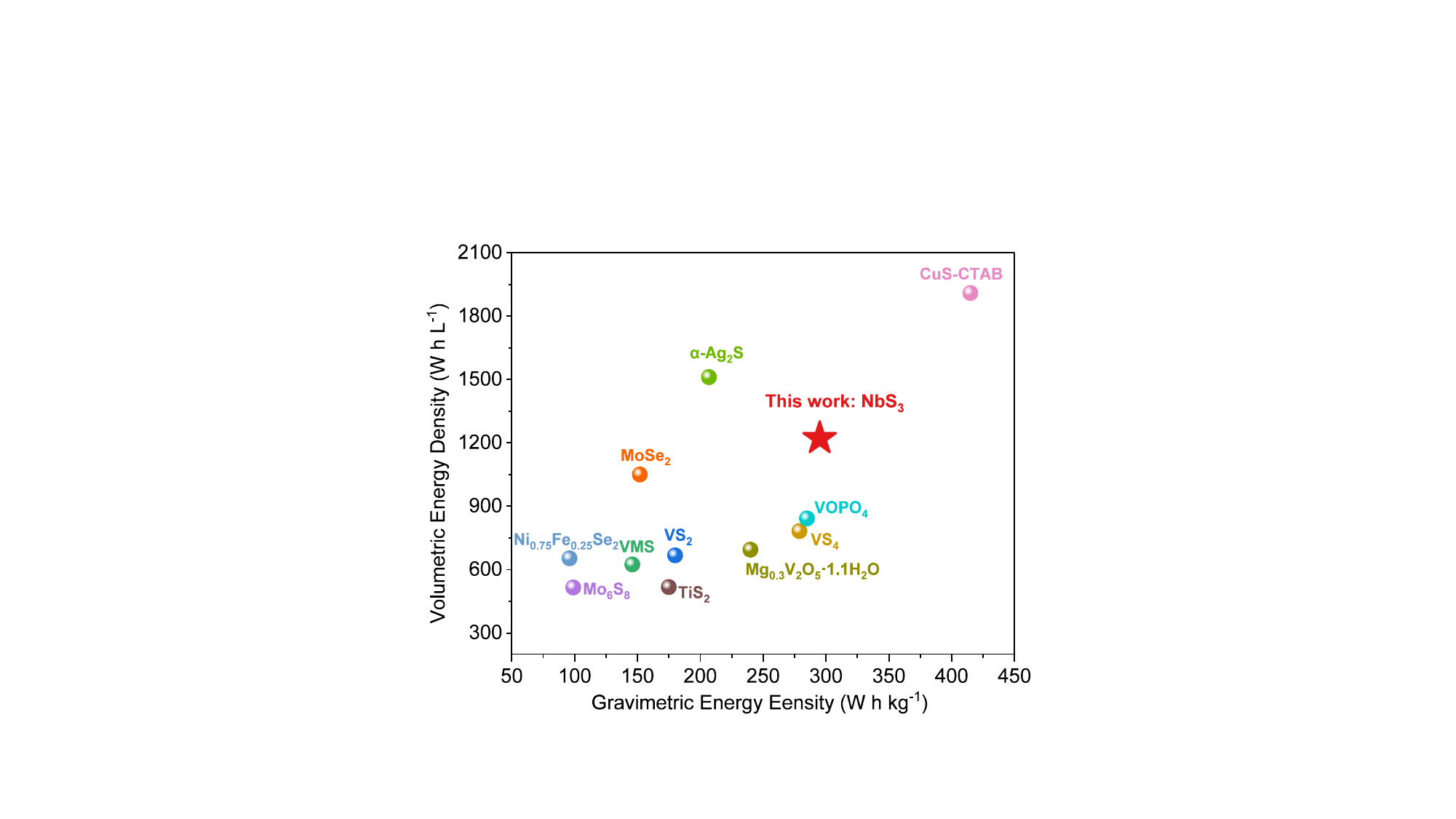

Supplement: Supplementary file 6 — Supporting File 6: advs74690‐sup‐0006‐Figure S5.pptx. [file ADVS-13-e74690-s003.pptx]

## Slide 1
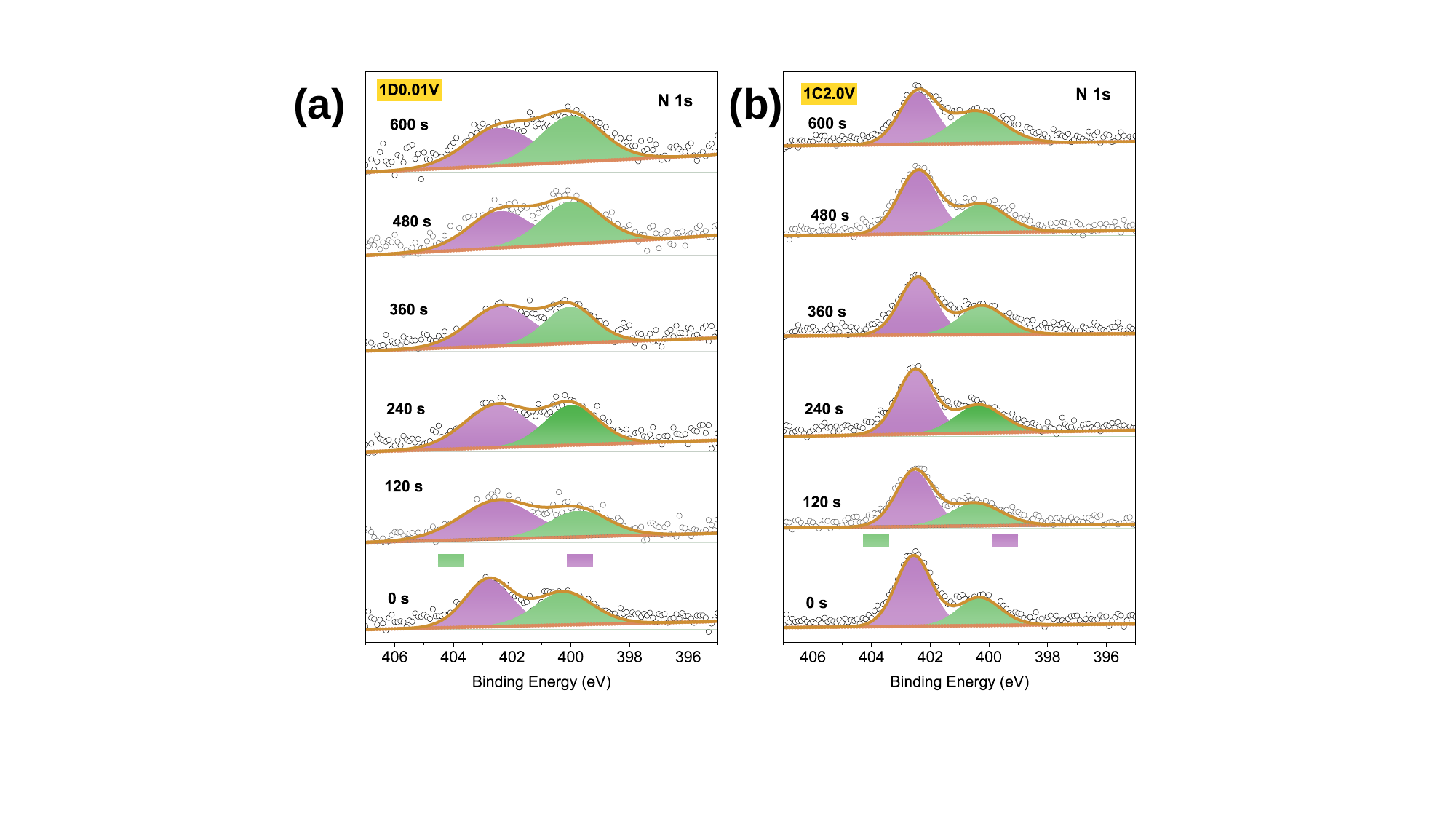

(a)
(b)

Supplement: Supplementary file 7 — Supporting File 7: advs74690‐sup‐0007‐Figure S6.pptx. [file ADVS-13-e74690-s006.pptx]

## Slide 1
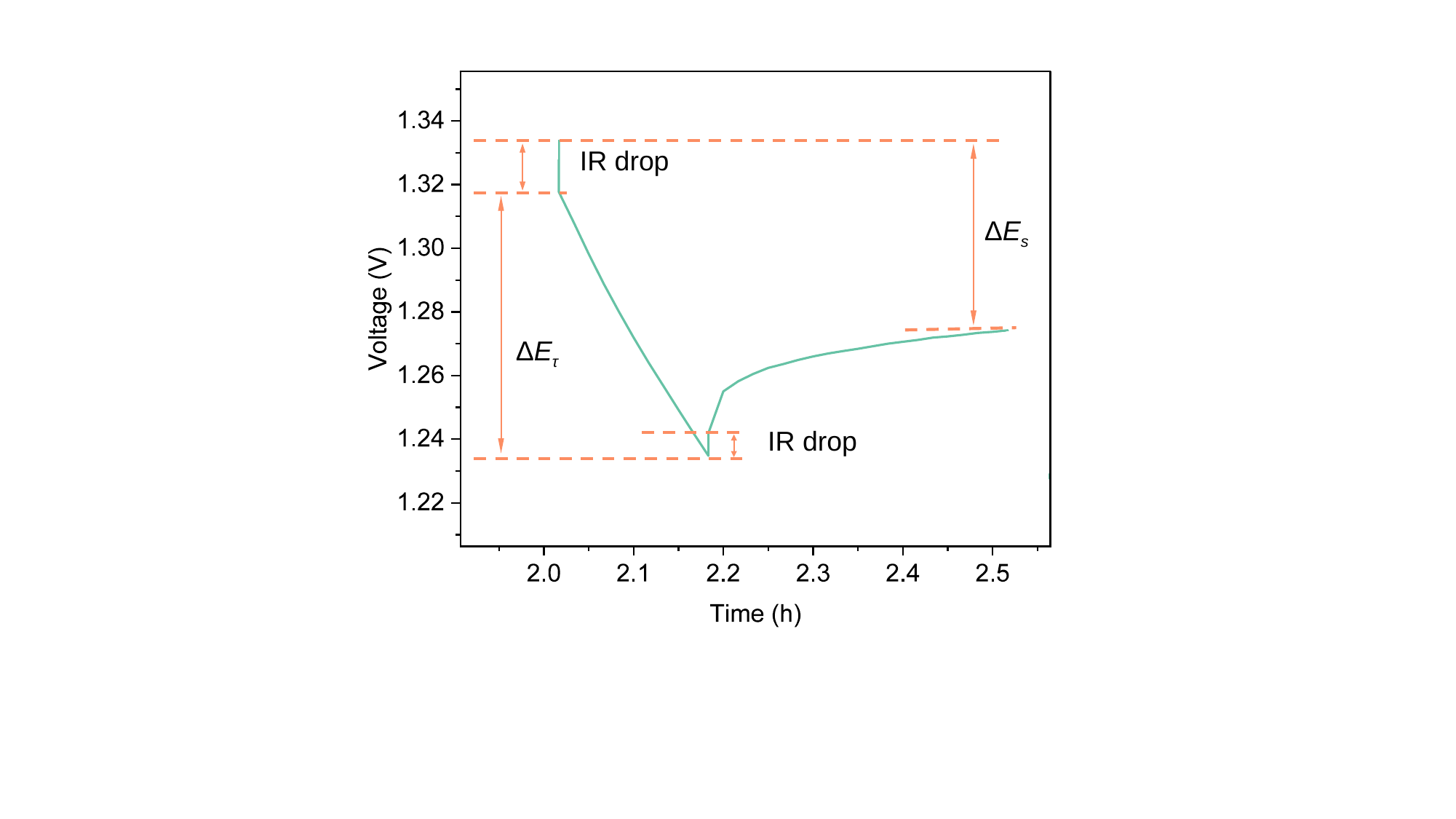

IR drop
ΔEs
ΔEτ
IR drop

Supplement: Supplementary file 8 — Supporting File 8: advs74690‐sup‐0008‐Figure S7.pptx. [file ADVS-13-e74690-s009.pptx]

## Slide 1
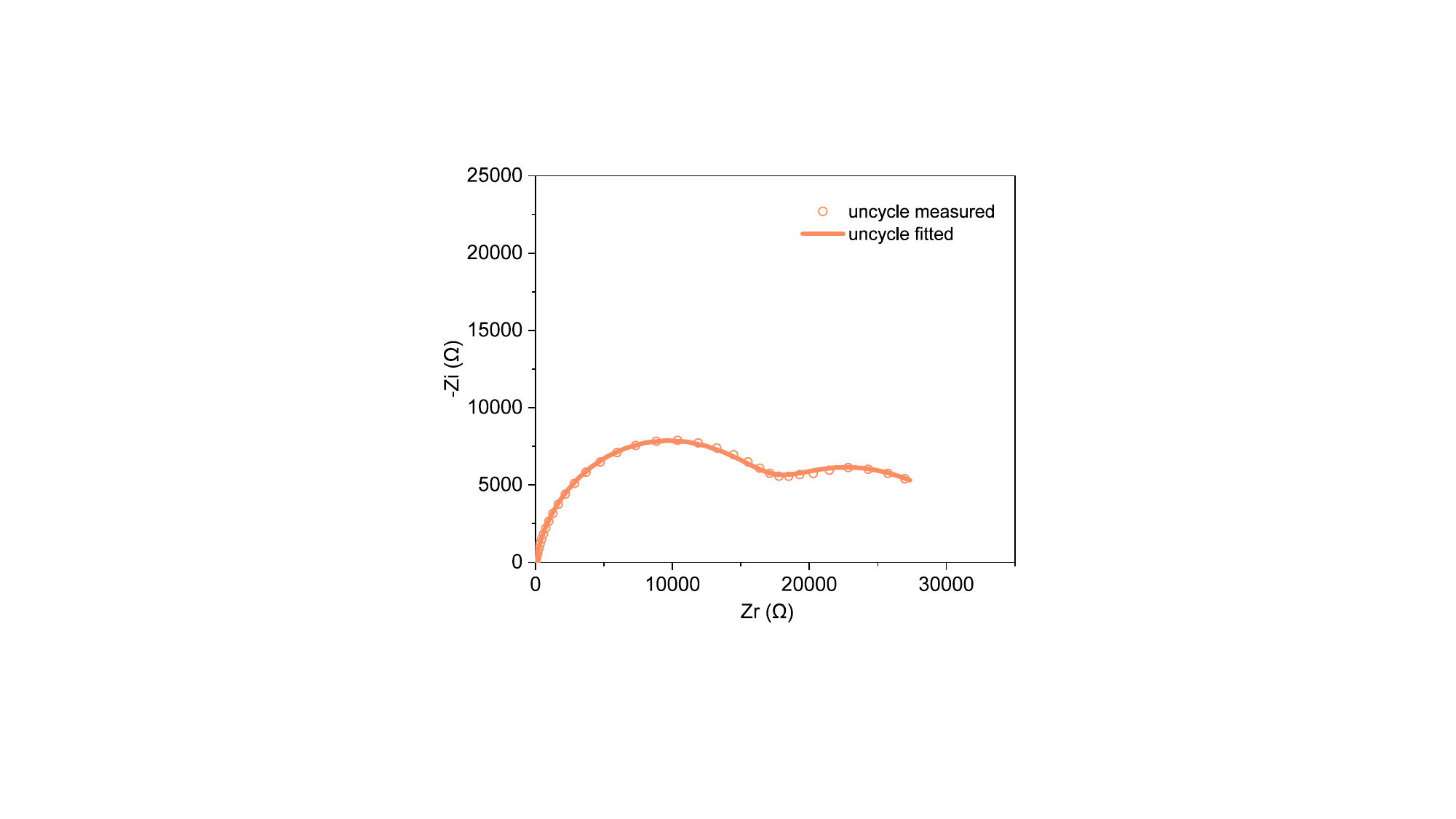

Supplement: Supplementary file 9 — Supporting File 9: advs74690‐sup‐0009‐Figure S8.pptx. [file ADVS-13-e74690-s008.pptx]
